# Effectiveness of peer counseling, social engagement, and combination interventions in improving depressive symptoms of community-dwelling Filipino senior citizens

**DOI:** 10.1371/journal.pone.0230770

**Published:** 2020-04-01

**Authors:** Rogie Royce Carandang, Akira Shibanuma, Junko Kiriya, Karen Rose Vardeleon, Edward Asis, Hiroshi Murayama, Masamine Jimba

**Affiliations:** 1 Department of Community and Global Health, Graduate School of Medicine, The University of Tokyo, Tokyo, Japan; 2 Institute of Gerontology, The University of Tokyo, Tokyo, Japan; 3 Childfam-Possibilities Psychosocial Services Co., Quezon City, Philippines; 4 Department of Global Studies, Faculty of Liberal Arts, Sophia University, Tokyo, Japan; Public Library of Science, UNITED KINGDOM

## Abstract

**Introduction:**

Little is known about community-based interventions for geriatric depression in low-resource settings. This study assessed the effectiveness of 3-month-duration interventions with peer counseling, social engagement, and combination vs. control in improving depressive symptoms of community-dwelling Filipino senior citizens.

**Methods:**

We conducted an open (non-blinded), non-randomized trial of senior citizens at risk for depression. Three different 3-month interventions included peer counseling (n = 65), social engagement (n = 66), and combination (n = 65) were compared with the control group (n = 68). We assessed geriatric depression, psychological resilience, perceived social support, loneliness, and working alliance scores at baseline and three months after the intervention. This trial was registered with ClinicalTrials.gov, identifier: NCT03989284.

**Results:**

Geriatric depression score over three months significantly improved in all intervention groups (control as reference). Significant improvements were also seen in psychological resilience and social support. Not all interventions, however, significantly improved the loneliness score. The combination group showed the largest effect of improving depressive symptoms (*d* = -1.33) whereas the social engagement group showed the largest effect of improving psychological resilience (*d* = 1.40), perceived social support (*d* = 1.07), and loneliness (*d* = -0.36) among senior citizens.

**Conclusion:**

At the community level, peer counseling, social engagement, and combination interventions were effective in improving depressive symptoms, psychological resilience, and social support among Filipino senior citizens. This study shows that it is feasible to identify senior citizens at risk for depression in the community and intervene effectively to improve their mental health. Further studies are required to target loneliness and investigate the long-term benefits of the interventions.

**Clinical trial:**

ClinicalTrials.gov: NCT03989284

## Introduction

Poor mental health is getting more common in low- and middle-income countries than in high-income countries due to lack of available resources and access to health services [1, 2]. In these countries, there is a large treatment gap for mental health care, with the majority of people with mental disorders receiving no or inadequate care [3]. Depression, for instance, is one of the most common mental disorders, and it affects physical health, social activities, and quality of life of senior citizens [4, 5]. Despite being a commonly studied mental disorder, very little is known about depression interventions conducted in low-resource settings.

Most depression interventions were conducted in developed countries and delivered in primary care [6–9] and home-based settings [10]. In the USA, most older adults were screened for depression and treated in primary care settings, yet often did not receive the recommended standard care for depression due to lack of care coordination and sustainable infrastructures [11]. A few studies evaluated home-based interventions in the community, and it led to better treatment acceptance [12] and fewer nursing home admissions and in-patient care days [13] among senior citizens. Mixed results, however, were obtained in community-based settings showing that interventions did improve the targeted outcomes (e.g., increases in physical therapy, training in certain skills) but did not alleviate depression [14].

Although evidence-based depression interventions exist, relatively few senior citizens seek care for mental health specialists [15]. Moreover, studies that examine the models used to deliver mental health services have been limited [15]. Given that senior citizens are less likely to seek specialty mental health services; community-based interventions have the potential to bring promising outcomes to engage this population. With appropriate interventions, depression is potentially reversible.

Based on existing literature, peer counseling and social engagement were found to be effective in alleviating depressive symptoms and improving the quality of life of community-dwelling senior citizens [16–19]. Peer counseling contributes to a positive social context that is necessary for recovering from mental disorders [20]. Peer-client relationships have the unique and distinct quality from that of professional-client relationships [21]. The unique benefits of peer relationships include empowerment, social network expansion, self-determination, and reduction of stigma [22, 23]. Clients in peer counseling programs showed improvement in mental health symptoms, greater satisfaction with health services, and feelings of greater autonomy [24]. Social engagement, on the other hand, shows that involvement in activities was beneficial for a healthy lifestyle and successful aging [19, 25]. Socially active senior citizens reported better health outcomes than their inactive counterparts, such as better physical functioning [26], higher cognitive functioning [27, 28], and lower mortality rates [29].

In the past, no studies have evaluated the individual and combined effects of peer counseling and social engagement concurrently in improving the mental health of community-dwelling senior citizens. We hypothesized that the combined intervention would be more effective than any single intervention in alleviating depressive symptoms. It could work together in either complementary or synergistic ways. This type of multilevel intervention is supported by the social-ecological perspective, which states that determinants at multiple levels (e.g., intrapersonal, interpersonal, organizational, community, and policy) interact to influence health outcomes and human behavior [30]. Following this, such multilevel interventions tend to produce more substantial and longer-lasting effects than any single-level interventions [30]. For instance, a multilevel intervention that combines peer counseling (an intrapersonal-level intervention) and social engagement program (an interpersonal and community-level intervention) is more likely to be more effective than either intervention alone. Several studies have documented multilevel interventions focusing on a variety of health conditions, including HIV, obesity, cancer, and cardiovascular disease [31].

In this study, we aimed to assess the effectiveness of 3-month-duration interventions with peer counseling, social engagement, and combined intervention vs. control in improving depressive symptoms among community-dwelling Filipino senior citizens. We compared the three interventions to determine their potential to become an evidence-based practice that can be implemented nationwide.

## Methods

This study was an open (non-blinded), non-randomized trial design focusing on community-dwelling senior citizens at risk for depression.

### Project overview and study setting

The project ENGAGE (Embracing and Nurturing Global Ageing) is community-based action research conducted in the City of Muntinlupa from 2017 to 2018. The project had three phases. In phase 1, we investigated the factors associated with depressive symptoms among community-dwelling Filipino senior citizens [32]. In phase 2, we trained senior volunteers for leadership and peer counseling [33]. In phase 3, we conducted community-based interventions to alleviate depressive symptoms among senior citizens. In this paper, we report on the results of Phase 3 of the study.

Muntinlupa is the southernmost city in the National Capital Region. The city had nine barangays and was classified as a highly urbanized city with a poverty incidence of 1.9% in 2012 [34]. In the Philippines, barangay is known as the smallest political unit, and it refers to a village or a community consisting of at least 2,000 inhabitants. The City of Muntinlupa also had one of the highest records of senior citizens, which account for 5.63% of its population [34].

### Study participants

From Phase 1 of the study, 575 of 1,021 (56.3%) community-dwelling senior citizens reported a depression score of 5 or more which indicated a tendency towards depression based on the 15-item Geriatric Depression Scale (GDS-15). The GDS-15 was used for depression screening [35–37], and it has an optimum cut-point of 5 or more. In the Philippines, however, no validation study of the GDS-15 has been reported yet. We then calculated the sample size for group allocation. We used Open Epi version 3.01 and based the following parameters from a meta-analysis [38] of the effects of outreach programs to depressed senior citizens in the community: effect size of 0.77, power of 90%, alpha set at 0.05 (two-sided) and expected dropout rate of 25%. We calculated at least 40 senior citizens per group. Considering the small sample size, we decided to increase the sample size to at least 60 senior citizens per group.

With help from Barangay Health Workers (BHWs), we did the recruitment purposively through home visits. The BHWs are the persons who have undergone training programs under an accredited government or non-government organization and render primary care services in the community [39]. They are vital in barangay health centers because they provide assistance and support to physicians, dentists, public health nurses, nutritionists, and midwives. Some of their responsibilities include collecting vital statistics, maintaining records and making reports, assisting in nutrition education, participating in community meetings, among others [39]. We involved them in the study because they are the first point of contact between the health care system and the rest of the community.

**Fig 1:**
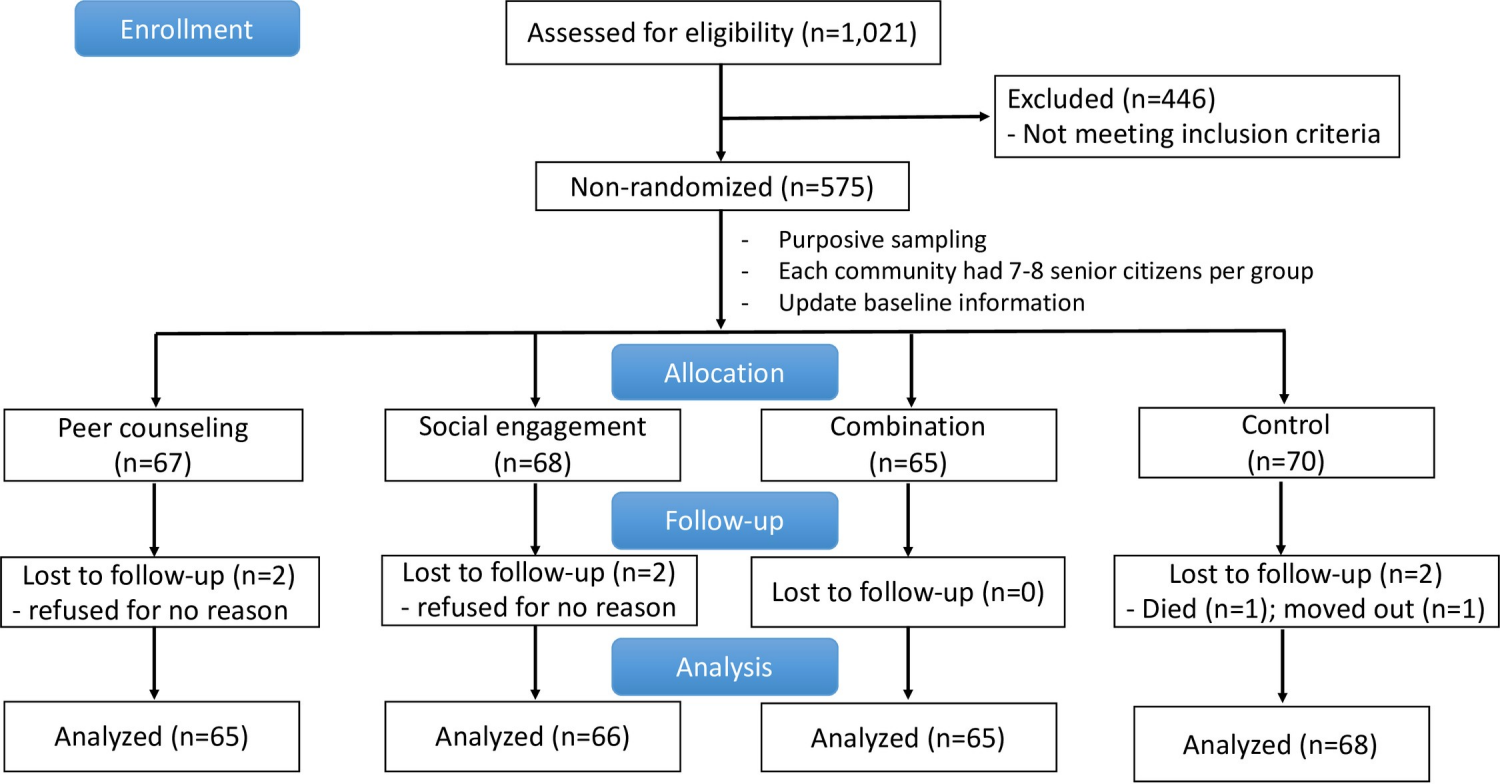
Flow chart of Phase 3 ENGAGE intervention study.

We used the pool of 575 senior citizens eligible for the study as our sampling frame. We allocated them non-randomly into four different groups: peer counseling (n = 67), social engagement (n = 68), combination (n = 65), and usual care control (n = 70) which accounted for a total of 270 participants (Fig 1). We made sure that each barangay had 7–8 senior citizens allocated per group to ensure the representativeness of the population. In case that a barangay did not have enough eligible senior citizens, we did the recruitment from its nearby barangay. We coordinated with barangay officials to provide a ‘pick-up and drop off service' for the senior citizens as they joined the weekly events at the Office for Senior Citizens Affairs (OSCA). We assigned at least two BHWs per barangay to take care of the senior citizens. We provided incentives (cash or goods) to all participants and BHWs who took part in the intervention.

We did the recruitment and allocation purposively for feasibility purposes. Only those who were present on the day of recruitment were included in the study. Senior citizens who were sleeping or those who went outside for health check-up or other personal reasons were not invited to participate. Senior citizens who agreed to participate were then assigned into different groups. For those who had difficulty walking outside, we assigned them to the peer counseling group. Otherwise, they can join either the social engagement or combined intervention group. It was essential to consider the functional ability or mobility of the senior citizens because the social events were held at OSCA, which is located in one barangay. The venue for the social event was on the second floor of the building, and senior citizens had to take the stairs to get there.

### Interventions

#### Peer counseling group

Peer counselors performed 1-hour home visits weekly to their assigned clients for three months. Peer counselors are senior volunteers who participated in Phase 2 of the study and underwent a 40-hour training for leadership and peer counseling. Details about peer counselors’ recruitment, supervision, training, and fidelity have been reported elsewhere [33]. The goals of the meetings were to establish a strong working alliance, identify a client-defined problem, encourage behavior change, and facilitate engagement with the community. At the initial visit, the peer counselor asked the clients what they would like to achieve from the meetings in order to establish a client-identified goal that they can work on together. Then, peer counselors accomplished home visitation weekly reports that they submitted and discussed during their monthly meetings.

Health providers (e.g., physician, psychologists, social workers, pharmacist) and BHWs met with the peer counselors once a month for an hour for supervision and collaboration. During meetings, the peer counselors reported on client's progress and shared impression and insights. The health providers and BHWs then provided guidance, reinforcement, and constructive feedback to continue the skills development of peer counselors.

#### Social engagement group

Senior citizens joined 3-hour weekly social events held at the OSCA Center for three months. We conducted the social events into two batches comprised of 30–35 senior citizens per batch. Each social event started with a prayer, with 15–20 minutes dancing, educational talk, group discussion/activity, interactive games, and karaoke. The health providers did the lecture while the peer counselors and BHWs assisted the group activity and interactive games. Older adults were not left on their own at the social event. Topics covered include healthy, active and successful aging; nutrition, physical activity, and functional ability; dealing with stress, loneliness, and depression; building confidence and resilience; problem-solving and decision-making; communication with others; and use of community resources. The key feature of the program was to enable senior citizens to expand their social network and promote active social participation within their respective communities. We developed the contents of the program and assessed the linguistic and cultural equivalence of our materials through a series of joint meetings with the OSCA staff.

#### Combination group

Senior citizens in this group underwent both peer counseling and social engagement interventions mentioned above. We designed the combination group to explore the additive or synergistic effect of peer counseling and social engagement on improving the mental health of senior citizens at risk for depression.

#### Control group

Senior citizens in this group had access to usual or standard care from health and aged care services that were usually available, including primary level care from barangay health centers and social welfare services delivered by the OSCA.

### Data collection

We used the Phase 1 data as a baseline measurement for depression screening, which was collected between October 1, 2017 and December 10, 2017. Then, we approached eligible senior citizens, updated their baseline scores, and recruited those who met the inclusion criteria to participate in the study between April 1, 2018 and April 12, 2018. Three months after the intervention, we performed outcome assessments. We did the follow-up assessment between August 1, 2018 to August 12, 2018. We did not blind the assessors to the senior citizens' group allocation. The same assessors (trained BHWs from Phase 1) collected the data through home visits [32].

### Variables and measurements

The instruments used in this study were described in detail in previous research [18, 32, 40]. We followed the WHO’s guideline for the process of translation and adaptation of instruments [41]. Two independent researchers (RRC and EA) translated the English version of the scales into Filipino separately and compared their output. Then, we asked the expert panel (two geriatric professionals and one psychologist) to identify and resolve the inadequate expressions or concepts of the translation, as well as any discrepancies between the forward translation and the previous versions of the scales. Then, another independent researcher translated the scales back to English. After that, we conducted pre-testing and cognitive interviewing among 30 senior citizens. We computed the reliability of the scales using Cronbach’s α.

#### Primary outcome

*Depressive symptoms*. We used the 15-item Geriatric Depression Scale (GDS-15) to measure the depressive symptoms of senior citizens. This scale contained fewer somatic items and was specially developed for use in geriatric patients [35–37]. The response options for all the items were ‘yes’ or ‘no’ and possible scores ranged from 0 to 15. The response to the five items dealing with positive well-being was reverse coded before obtaining the total GDS-15 score. The five items were:

Q1. Are you basically satisfied with your life?
Q5. Are you in good spirits most of the time?
Q7: Do you feel happy most of the time?
Q11: Do you think it is wonderful to be alive?
Q13: Do you feel full of energy?

A score of 5 or more indicated a tendency towards depression. The validity and reliability of GDS-15 have been supported through both clinical practice and community-based research [42, 43]. The Cronbach’s α for this study was 0.84.

#### Secondary outcomes

*Psychological resilienc*. We used the 12-item Resilience Appraisal Scale (RAS-12) to measure senior citizens’ psychological resilience. The scale consisted of three parts of coping skills such as problem-solving, emotional regulation, and social support seeking [44]. Using a five-point Likert scale ranging from 1 (strongly disagree) to 5 (strongly agree), senior citizens indicated the degree of applicability of each statement to them. The total RAS-12 score ranged from 12 to 60, with a higher score indicating higher perceived psychological resilience. The Cronbach’s α for this study was 0.93.

*Perceived social support*. We used the 10-item Duke Social Support Index (DSSI-10) to assess the senior citizens’ perceived social support. The scale measured two essential concepts related to social support, such as social interaction and satisfaction with social support [45]. The possible score ranged from 10 to 30, with higher scores indicating a higher level of perceived social support among senior citizens. The Cronbach's α for this study was 0.82.

*Loneliness*. We used the 8-item UCLA Loneliness Scale (ULS-8) to measure senior citizens' loneliness. The scale [46] employed a four-point Likert scale with values ranging from 0 (never) to 3 (often), and the total score ranged from 8 to 32. The response to ‘I can find companionship when I want it’ and ‘I am an outgoing person’ were reverse coded before obtaining the total score for all eight items. There was no cut-off score identified to define loneliness. However, a higher score on this scale indicated more intense feelings of loneliness. The Cronbach’s α for this study was 0.82.

*Working alliance*. Working alliance is the trust between a client and peer counselor, which enables them to work together for the benefit of the client. It is the bond that helps a client have complete faith in their counselor [47]. We measured their working alliance using the Working Alliance Inventory-Short Form (WAI-SF). The WAI-SF measures three domains of the therapeutic alliance: (1) agreement between client and peer counselor on the goals of the treatment (Goal); (2) agreement between client and peer counselor about the tasks to achieve these goals (Task); and (3) the quality of bond between the client and peer counselor (Bond) [48]. Peer counselors and clients completed the WAI-SF on their first and last meeting. Only the peer counseling and combination groups used this questionnaire. The WAI-SF scores ranged from 12–84, with higher scores indicating a stronger bond and agreement on tasks and goals. The Cronbach's α for this study was 0.85 and 0.88 for the peer counselors and clients, respectively.

*Satisfaction with the social engagement program*. We evaluated senior citizens' satisfaction with the program using rating scale questions, which displayed a scale of either from ‘1 to 5' or ‘0 to 10'. Senior citizens in the social engagement and combination interventions were asked to select the numerical value on the scale, which represents their response best. The higher the numerical point, the more they strongly agree with the statement and vice versa. We performed face validity testing by asking a group of mental health experts before the administration of the rating scale.

### Data analysis

We performed comparisons across three intervention groups using generalized estimating equations and analysis of variance (whichever is applicable) for continuous variables and chi-squared tests for categorical variables. We reported the net mean change from baseline between intervention groups (using the control group as the reference) and its 95% confidence interval (CI) for continuous outcomes as well as the effect size (Cohen’s *d*) of each intervention. The net mean change represented the absolute change in the score while the effect size represented the relative change. We also used linear regression to measure the magnitudes of the association between change in GDS-15 scores and change in WAI-SF scores and its subscales. We set the level of significance to 0.05 (two-tailed) and performed statistical analyses using Stata 13.1 (StataCorp, College Station, TX, USA).

### Ethical considerations

The Project ENGAGE was approved by the University of Tokyo Research Ethics Committee (SN 11641) and the University of the Philippines-Manila Research Ethics Board (UPMREB 2017-312-01). We ensured the confidentiality of the senior citizens’ responses and strictly protected their privacy as no personally identifiable information was used in this study. Also, we secured written informed consents before the intervention, and all participation was voluntary. We obtained all required permits and approvals as applied to foreign researchers.

This study was registered retrospectively in ClinicalTrials.gov with identifier NCT03989284. This study is action research that focused on improving the depressive symptoms of community-dwelling senior citizens. We were not aware of a suitable registry for such action research with an open (non-blinded), non-randomized trial design conducted in the Philippines before data collection. However, we confirm that all ongoing and related trials for this intervention are registered.

## Results

Of the 1,021 senior citizens, 575 (56.3%) were suggestive of having depression based on the GDS-15 score. The dropout rate for this study was low and comparable across the four intervention groups (3.0% for peer counseling; 2.9% for social engagement; 0.0% for combination; and 2.9% for control). The reasons for dropout were: moved residence (n = 1), death (n = 1), and refused for no reason (n = 4). Two hundred sixty-four senior citizens (97.8%) completed the 3-month follow-up assessment. No adverse events occurred during the study.

The senior citizens’ mean age was 68.3 years [standard deviation (SD) 6.1], and 187 (70.8%) were women. All of them were at risk for depression (based on screening test). We did not observe any statistically significant differences in baseline mental health outcomes and socio-demographic characteristics across the intervention groups (Table 1).

**Table 1:** Characteristics of community-dwelling senior citizens at risk for depression at baseline (*N* = 264).

Characteristics	Peer counseling (n = 65)	Social engagement (n = 66)	Combination (n = 65)	Control (n = 68)	*p*-value
Age in years, mean (SD)	68.0 (5.7)	68.8 (5.9)	68.2 (5.4)	68.3 (7.2)	0.406
Sex, n (%)					0.891
Men	19 (29.2)	19 (28.8)	17 (26.2)	22 (32.4)	
Women	46 (70.8)	47 (71.2)	48 (73.9)	46 (67.7)	
Marital status, n (%)					0.072
Married/ Remarried	28 (43.1)	32 (48.5)	31 (47.7)	33 (48.5)	
Never married	5 (7.7)	3 (4.6)	5 (7.7)	12 (17.7)	
Separated	3 (4.6)	0 (0.0)	1 (1.5)	4 (5.9)	
Widowed	29 (44.6)	31 (47.0)	28 (43.1)	19 (27.9)	
Education, n (%)					0.328
No education	1 (1.5)	3 (4.6)	0 (0.0)	0 (0.0)	
Primary	38 (58.5)	36 (54.6)	41 (63.1)	38 (55.9)	
Secondary/Tertiary	26 (40.0)	27 (40.9)	24 (36.9)	30 (44.1)	
Monthly income, n (%)					0.990
No income	46 (70.8)	48 (72.7)	49 (75.4)	48 (70.6)	
Poor income	16 (24.6)	16 (24.2)	13 (20.0)	17 (25.0)	
Average/Good income	3 (4.6)	2 (3.0)	3 (4.6)	3 (4.4)	
Pension, n (%)					0.459
Have	28 (43.1)	37 (56.1)	30 (46.2)	31 (45.6)	
Do not have	37 (56.9)	29 (43.9)	35 (53.9)	37 (54.4)	
Self-rated health, n (%)					0.930
Good/Very good	12 (18.5)	16 (24.2)	14 (21.5)	14 (20.6)	
Fair	36 (55.4)	30 (45.5)	30 (46.2)	35 (51.5)	
Bad/Very bad	17 (26.2)	20 (30.3)	21 (32.3)	19 (27.9)	
Chronic diseases, n (%)					0.869
Have	59 (90.8)	58 (87.9)	57 (87.7)	62 (91.2)	
Don’t have	6 (9.2)	8 (12.1)	8 (12.3)	6 (8.8)	
Living arrangement, n (%)					0.521
Alone	6 (9.2)	9 (13.6)	5 (7.7)	10 (14.7)	
Living with others	59 (90.8)	57 (86.4)	60 (92.3)	58 (85.3)	
Smoking, n (%)					0.542
Never-smoker	51 (78.5)	54 (81.8)	47 (72.3)	50 (73.5)	
Ex-/ Current-smoker	14 (21.5)	12 (18.2)	18 (27.7)	18 (26.5)	
Drinking alcohol, n (%)					0.527
Non-drinker	48 (73.9)	55 (83.3)	52 (80.0)	56 (82.4)	
Occasional/ Daily drinker	17 (26.2)	11 (16.7)	13 (20.0)	12 (17.7)	
GDS-15 score, mean (SD)	7.1 (2.0)	7.6 (2.3)	7.8 (2.5)	7.0 (1.7)	0.114
RAS-12 score, mean (SD)	45.4 (5.2)	44.8 (5.3)	45.4 (5.4)	45.3 (3.8)	0.896
DSSI-10 score, mean (SD)	21.4 (3.4)	21.4 (3.4)	21.0 (3.6)	22.2 (3.1)	0.224
ULS-8 score, mean (SD)	8.3 (3.5)	8.6 (3.5)	9.0 (3.9)	7.6 (4.2)	0.157

*GDS-15* 15-item Geriatric Depression Scale; *RAS-12* 12-item Resilience Appraisal Scale; *DSSI-10* 10-item Duke Social Support Index; *ULS-8* 8-item UCLA Loneliness Scale; *SD* Standard deviation.

Table 2A and 2B summarize the effect of interventions on the mental health outcomes of senior citizens at risk for depression from baseline and at three month- follow-up, using the control group as reference. There was a significant net mean change, with improvement in depressive symptoms, psychological resilience, and perceived social support over three months across all intervention groups. Significant improvement in loneliness score was obtained only in the social engagement group. Moreover, the combination group showed the largest effect of improving depressive symptoms (*d* = -1.33) whereas the social engagement group showed the largest effect of improving psychological resilience (*d* = 1.40), perceived social support (*d* = 1.07), and loneliness (*d* = -0.36) among community-dwelling Filipino senior citizens.

**Table 2:** Effects of intervention on the mental health outcomes of community-dwelling senior citizens at risk for depression.

A									
		Mean (SD)			
		Peer counseling (n = 65)	Social engagement (n = 66)	Combination (n = 65)	Control (n = 68)
GDS-15 score	Pre	7.1 (2.0)	7.6 (2.3)	7.8 (2.5)	7.0 (1.7)
	Post	5.0 (3.0)	4.0 (2.3)	3.5 (2.5)	6.3 (3.1)
RAS-12 score	Pre	45.4 (5.2)	44.8 (5.3)	45.4 (5.4)	45.3 (3.8)
	Post	48.6 (5.1)	52.9 (6.2)	51.0 (6.0)	43.4 (5.2)
DSSI-10 score	Pre	21.4 (3.4)	21.4 (3.4)	21.0 (3.6)	22.2 (3.1)
	Post	23.9 (3.3)	24.1 (3.2)	23.2 (3.3)	20.3 (3.1)
ULS-8 score	Pre	8.3 (3.5)	8.6 (3.5)	9.0 (3.9)	7.6 (4.2)
	Post	9.4 (4.0)	9.5 (4.8)	11.2 (4.3)	10.3 (4.2)
B									
	Peer counseling (n = 65)			Social engagement (n = 66)			Combination (n = 65)		
	Net mean change^a^ (95% CI)	*p*-value	Effect size^b^	Net mean change^a^ (95% CI)	*p*-value	Effect size^b^	Net mean change^a^ (95% CI)	*p*-value	Effect size^b^
GDS-15 score	-1.4 (-2.5, -0.31)	0.012	-0.44	-2.9 (-3.8, -2.0)	<0.001	-1.10	-3.6 (-4.5, -2.7)	<0.001	-1.33
RAS-12 score	5.0 (2.7, 7.4)	<0.001	0.72	9.9 (7.6, 12.3)	<0.001	1.40	7.6 (5.3, 9.9)	<0.001	1.13
DSSI-10 score	4.4 (2.8, 6.0)	<0.001	0.92	4.5 (3.1, 6.0)	<0.001	1.07	4.1 (2.5, 5.7)	<0.001	0.87
ULS-8 score	-1.7 (-3.5, 0.0)	0.056	-0.33	-1.8 (-3.6, -0.1)	0.038	-0.36	-0.6 (-2.3, 1.2)	0.532	-0.11

*GDS-15* 15-item Geriatric Depression Scale; *RAS-12* 12-item Resilience Appraisal Scale; *DSSI-10* 10-item Duke Social Support Index; *ULS-8* 8-item UCLA Loneliness Scale. *SD* Standard deviation; *CI* Confidence interval

^a^ Net mean change from baseline using control group as reference

^b^ This effect size (Cohen’s *d)* is a standardized measure of the difference in differences between the intervention and control group in standard-deviation units and the minus (-) sign indicates the negative direction of the effect which in this case reduction of depressive symptoms and loneliness; Adjusted for age, sex, marital status, education, monthly income, pension, self-rated health, chronic diseases, living arrangement, smoking, and drinking.

Table 3 shows the magnitude of association between change in GDS-15 scores and change in WAI-SF scores and its subscales (bond, task, goal) over three months. In the peer counseling group, those who had stronger working alliance were negatively associated with higher level of depressive symptoms (peer ratings: β = -0.14; 95% CI = -0.22, -0.06; client ratings: β = -0.27; 95% CI = -0.42, -0.11). We observed the same pattern in the combination group (peer ratings: β = -0.19; 95% CI = -0.28, -0.11; client ratings: β = -0.15; 95% CI = -0.22, -0.08). The combination group, however, showed a larger effect of improving working alliance (peer ratings: *d* = 1.66; client ratings: *d* = 1.84) as compared with the peer counseling group (peer ratings: *d* = 0.52; client ratings: *d* = 0.50).

**Table 3:** Association between change in GDS-15 scores and change in WAI-SF scores and its subscales (bond, task, goal).

	Peer counseling (n = 65)			Combination (n = 65)		
	GDS-15 scores			GDS-15 scores		
Measures	β (95% CI)	*p*-value	Effect size^a^	β (95% CI)	*p*-value	Effect size^a^
**Peer ratings**						
Total WAI-SF score	-0.14 (-0.22, -0.06)	0.001	0.52	-0.19 (-0.28, -0.11)	<0.001	1.66
Bond subscale	-0.25 (-0.47, -0.04)	0.022	0.55	-0.33 (-0.51, -0.16)	<0.001	1.34
Task subscale	-0.25 (-0.44, -0.06)	0.013	0.54	-0.33 (-0.56, -0.11)	0.005	1.82
Goal subscale	-0.37 (-0.59, -0.15)	0.001	0.14	-0.36 (-0.58, -0.14)	0.002	0.77
**Client ratings**						
Total WAI-SF score	-0.27 (-0.42, -0.11)	0.001	0.50	-0.15 (-0.22, -0.08)	<0.001	1.84
Bond subscale	-0.48 (-0.76, -0.19)	0.002	0.98	-0.19 (-0.36, -0.03)	0.024	1.44
Task subscale	-0.13 (-0.47, 0.21)	0.451	0.34	-0.36 (-0.50, -0.22)	<0.001	1.22
Goal subscale	-0.19 (-0.45, 0.06)	0.131	0.47	-0.12 (-0.29, 0.05)	0.176	1.31

*GDS-15* 15-item Geriatric Depression Scale; *WAI-SF* Working Alliance Inventory-Short Form; β Standardized beta adjusted for age, sex, marital status, education, and monthly income

^a^ This effect size (Cohen’s *d)* is a standardized measure of the difference between before and after the intervention in standard-deviation units.

Table 4 shows the evaluation of social engagement program. Both social engagement and combined intervention groups completed the questionnaire at the end of the program. The results indicated a high satisfaction level among senior citizens (average 9.9 on a 10-point scale) and with specific aspects of the program, which include topics chosen, time allocation, materials used, quality of invited speakers, and event facility (range of item means = 4.4–4.8 on a 1–5 scale). Moreover, attitudinal responses revealed that senior citizens found the social events to be relevant, useful, and easy to understand which led to an increased amount of knowledge and a change in their attitude (range of item means = 9.8–9.9 on a 0–10 scale).

**Table 4:** Evaluation of social engagement program.

Measure	Possible range of scores	Social engagement (n = 66)	Combination (n = 65)	*p*-value
**Depressed senior citizens’ satisfaction**		Mean (SD)	Mean (SD)	
Topics chosen	1–5	4.5 (0.7)	4.7 (0.5)	0.054
Time allocation	1–5	4.6 (0.6)	4.5 (0.6)	0.394
Materials used	1–5	4.7 (0.6)	4.6 (0.5)	0.261
Quality of invited speakers	1–5	4.6 (0.7)	4.4 (0.7)	0.103
Event facility	1–5	4.8 (0.4)	4.7 (0.6)	0.064
Clarity and understandability	0–10	9.9 (0.7)	9.9 (0.3)	0.750
Amount of new knowledge gained	0–10	9.9 (0.6)	9.8 (0.5)	0.409
Relevance and usefulness	0–10	9.8 (0.7)	9.8 (0.4)	0.892
Amount of attitude change	0–10	9.8 (0.7)	9.9 (0.3)	0.439
Overall satisfaction	0–10	9.9 (0.3)	9.9 (0.5)	0.316

*SD* Standard deviation

## Discussion

In this study, geriatric depression score over three months significantly improved in all intervention groups (control as reference). Significant improvements were also seen in psychological resilience and perceived social support. Not all interventions, however, significantly improved the loneliness score. On the other hand, the combined intervention group showed the largest effect of improving depressive symptoms (*d* = -1.33) whereas the social engagement group showed the largest effect of improving psychological resilience (*d* = 1.40), perceived social support (*d* = 1.07), and loneliness (*d* = -0.36) among community-dwelling Filipino senior citizens.

Peer counseling has improved the mental health outcomes of senior citizens after participating in the program. It showed a large effect of improving perceived social support (*d =* 0.92), moderate effect on psychological resilience (*d =* 0.72) and depressive symptoms (*d =* -0.44), and small effect on loneliness (*d* = -0.33). We attributed the success of the peer counseling program for two reasons. First, a stronger working alliance between client and peer counselor was associated with a lower level of depressive symptoms. We measured their working alliance concerning their ability to connect emotionally, define goals, and work collaboratively to specific tasks to reach their goals. According to a recent meta-analysis, a moderate but reliable association was shown between good working alliance and positive therapy outcome [49]. This finding is in agreement with our study. Therefore, a strong working alliance is an essential element in counseling. Second, the involvement of health providers and BHWs may have provided a certain level of quality and accountability, which led to the credibility of the peer counselors in this program. This innovative model of depression care delivery was previously reported in the USA [18], and we adopted and modified it in our study using various health providers.

Social engagement also improved the mental health outcomes of Filipino senior citizens. It showed a large effect of improving depressive symptoms (*d* = -1.10), psychological resilience (*d* = 1.40) and perceived social support (*d* = 1.07), and showed a small effect on loneliness (*d* = -0.36). Our findings, however, did not match the longitudinal studies conducted in the USA and Korea. For instance, Glass et al. [50] stated that social engagement was independently associated with depressive symptoms at a specific point in time and that longitudinal association was seen only among Americans who were not depressed at baseline. In their study, they excluded senior citizens who exhibited elevated depression scores at baseline and follow-up in their analysis [50], and this might account for the difference in the results of our study. Meanwhile, the social gathering had a positive effect on depressive symptoms over time in non-depressed Korean senior citizens at baseline, and no such effect existed among those who were already depressed [51]. These findings were not in agreement with our study. In their study, they did not consider participation frequency and quality of social activities [51], and this limitation might account for the difference in the results of our study.

As expected, the combined intervention showed a large effect of improving depressive symptoms (*d* = -1.33), psychological resilience (*d* = 1.13), perceived social support (*d* = 0.87), and working alliance between client (*d* = 1.84) and peer counselor (*d* = 1.66). However, it showed a small effect of improving loneliness (*d* = -0.11). The combined intervention showed the largest effect of improving depressive symptoms, which supported our hypothesis. This is the first study that reported the effectiveness of a combined intervention in improving depressive symptoms. Our results indicated the benefit of the three-month duration of combined intervention in improving mental health outcomes of Filipino senior citizens.

All interventions seemed to have a small effect of improving loneliness among Filipino senior citizens. Senior citizens could be less depressed but remained lonely after the intervention. According to Hawkley and Cacioppo [52], loneliness is not merely being alone. It is a distressing feeling that accompanies the perception that one has unmet social needs [52,53]. Senior citizens, in this study, might have been suffering from chronic perceived isolation, which requires longer intervention time and a more tailored approach.

Both the peer counseling and social engagement programs were well accepted by the senior citizens as exemplified by the low dropout rates across the three intervention groups and by the positive ratings obtained from the survey. We adopted an innovative model of depression care delivery [18] and proved that collaboration among academic, social welfare and health sectors in coordinating care is feasible in a low-resource setting.

### Strengths and limitations

To our knowledge, this is the first intervention study that evaluated the effects of peer counseling, social engagement, and combined intervention concurrently in improving depressive symptoms of community-dwelling senior citizens. A few intervention studies have evaluated peer counseling [16–18] or social engagement [19] singly, without differentiating their individual effects.

We acknowledged several limitations of this study. First, senior citizens in this study were purposively recruited based on their GDS-scores and physical health. The used of the cutoff score (GDS ≥ 5) to select the 575 from the 1,021 senior citizens and the assignment of participants with poor physical health in the peer counseling group can potentially lead to misclassification. Second, the senior citizens were not randomly allocated to either intervention or control groups. The lack of randomization can result in selection bias, potential confounding, and larger treatment effects. Despite non-randomization, senior citizens across the three intervention groups and the control group showed homogenous profiles of baseline mental health outcomes and socio-demographic characteristics. Third, we included standardized effect sizes because sometimes they can be useful, but categorizing effect sizes into small, medium, and large carries with it all of the disadvantages of categorization in general. Fourth, we used GDS-15 as a screening tool for clinical depression without further assessment, which may limit the generalizability of the findings. Senior citizens in this study were only at risk or suggestive of having depression. Fifth, some of the measures such as RAS-12, DSSI-10, and ULS-8 were adapted from previous studies [32, 40, 54,55], and have not been validated in the Philippine context. However, we did forward and back translations carefully, performed face-validity testing by asking the expert panel, pretested the questionnaires, and confirmed their reliability. Finally, the follow-up period was only three months. We have confirmed the short-term but not the long-term benefits of the interventions.

## Conclusions

At the community level, peer counseling, social engagement, and combination interventions were effective in improving depressive symptoms, psychological resilience, and social support among Filipino senior citizens. This study shows that it is feasible to identify senior citizens at risk for depression in the community and intervene effectively to improve their mental health. Other resource-limited communities can learn from the Philippines' experience and treat their senior citizens at risk in similar ways. Further studies are required to target loneliness and investigate the long-term benefits of the interventions.

## Supporting information

S1 File TREND checklist. (PDF)

S2 File Research proposal. (DOCX)

S3 File UTokyo ethics approval. (PDF)

S4 File UPMREB ethics approval. (PDF)
