# Supplementary material for: Effectiveness of peer counseling, social engagement, and combination interventions in improving depressive symptoms of community-dwelling Filipino senior citizens
Source: PLoS One. 2020 Apr 1;15(4):e0230770. doi: 10.1371/journal.pone.0230770 (PMC7112231; doi:10.1371/journal.pone.0230770)
Supplement: S2 File — (DOCX) [file pone.0230770.s002.docx]

**Project ENGAGE: An action research towards improving the psychological wellbeing of community-dwelling seniors in the Philippines**

Table of contents

Table of contents i

Abbreviations ii

1. Introduction 4

1.1 Aging: a global phenomenon 4

1.2 The Filipino elderly 4

1.3 The Filipino family 5

1.4 Concerns of the elderly in the Philippines 5

1.5 Situational analysis …………………………………………………………… 8

1.6 Problem analysis………………………………………………………..............9

1.7 Aging and mental health……………………………………………………….10

2. Project description………………………………………...……………………….....11

2.1 Phase 1…………………………………………………………………………12

2.2 Phase 2…………………………………………………………………………16

2.3 Phase 3…………………………………………………………………………19

2.4 Project timeline…………………………………………………………….......23

2.5 Ethical considerations………………………………………………………….24

2.6 References…………………………………………………………………...... 29

Abbreviations

AARP American Association of Retired Persons

BHW Barangay Health Worker

BSNOH Baseline Survey for the National Objectives for Health

CHO City Health Office

DOH Department of Health

DSWD Department of Social Welfare and Development

EFW Elderly Filipino Week

ENGAGE Embracing and Nurturing Global Ageing

GDS Geriatric Depression Scale

HALE Healthy Life Expectancy

LHB Local Health Board

LGU Local Government Unit

NCR National Capital Region

OSCA Office of Senior Citizen Affairs

PhilHealth Philippine Health

PopCom Commission on Population

QOL Quality of Life

WHO World Health Organization

# INTRODUCTION

# Aging: a global phenomenon

The world is aging. Most people can expect to live into their 60s and beyond (United Nations Department of Economic and Social Affairs 2007). Europe and Japan were among the first places to experience population aging. However, the most dramatic change is now occurring fastest in low- and middle-income countries (World Health Organization 2012). The changes are dramatic and the demographic shifts have profound implications to the society.

The main reason for population aging is the improvement of survival chances. For instance, low and middle-income countries experienced large reductions in mortality at younger ages and from infectious diseases (Bloom 2011). High-income countries, on the other hand, continue to increase their life expectancy mainly due to declining mortality of older people (Christensen et al. 2009).

Increasing longevity is not always accompanied by an extended period of good health (Beard et al. 2016). This is specifically true among people from disadvantaged backgrounds. Poor countries have the fewest opportunities and resources to rely on. Hence, more likely to have the poorest health (Lindmeier & Brunier 2015). The government must then ensure policies that will inspire, inform, and promote good health. Older people must continue to participate in the society and make significant contribution to nation building. In so doing, they can achieve a more dignified and meaningful life.

**The Filipino elderly**

***Increasing number of senior citizens***

In the Philippines, people who are regarded as senior citizen are those aged 60 years old and over. They made up 6.8% of the 92.1 million household population in 2010, higher than the 6.0% recorded in 2000 (Ericta 2012). Among the senior citizens, females (55.8%) outnumbered the males (44.2%). The National Statistics Office projects that by 2030, older people will make up around 11.5% of the total population. Thus, the Philippines is currently on the boundary of a demographic transition stage of an aging population.

According to the World Health Organization (WHO), Filipinos have an average lifespan of 68.41 years. This is almost three years shorter than the global average of age 71.4. The Healthy Life Expectancy (HALE) at birth was 63.9 for women and 58.6 for men. HALE at age 60 was 14.3 years for women and 11.9 years for men (World Population Prospects 2010).

Old-age dependency ratio has increased from 5.6% in 1970 to 7% in 2010 (Albert 2012), indicating that the number of working persons needed to support a retired person hardly changed from 6 in 1970 to 7 in 2010. In general, the Philippine population is characterized by growing proportion in the working ages and a slightly increasing elderly population.

**The Filipino family**

Filipinos are generally known to take care of their elderly family members and not leave them to the care of an institution or other people (AARP Real Possibilities 2014). A study conducted by the American Association of Retired Persons (AARP) noted that 42% of Asians (with Filipinos ranking second, next to Chinese) are direct caregivers to the aging parents and relatives. The Filipino value of *utang na loob* (debt of gratitude) makes the children care for their parents at their old age. This is one way to show gratitude for giving them life and for caring and rearing them while they were young (Andres 2002).

Elderly people can find joy whenever they are spending time with their family and grandchildren. However, they feel some degree of anxiety concerning their declining physical and cognitive health as they age. This is aggravated by increasing migration of children to other countries. Remittances from children working abroad are assumed to improve economic support to their parents but the effect in terms of provision of physical care is underestimated. In addition, the increasing migration of young women to other countries to work as caregivers reduces the number of potential familial caregivers of the elderly in their own families (Abejo 2004).

**Concerns of the elderly in the Philippines**

***Security in old age***

Filipino elderly count among the poorest sectors in the Philippines. The National Statistics Coordination Board (NSCB) said the sector where the senior citizens belong has the sixth highest poverty incidence among the eight basic sectors in the Philippines with 15.1% in 2003 and 16.2% in 2006 (Virola 2011). Poverty is then perceived as an impediment to a secured old age (Carlos 1999).

The current pension system in the Philippines requires careful consideration and evaluation. Pensions have remained largely stagnant in recent years, and health expenditures which rise, as people age, are barely subsidized by the government. Only a small percentage of the elderly are actually covered by pensions (Ubalde 2011). The government, on the other hand, offers welfare services such as homes for the aged and Senior Citizen centers to better address the struggle of the Filipino elderly. However, these nursing homes cater only to the abandoned, needy, neglected or unattached elderly (Carlos 1999). These homes are generally small and overpopulated. Majority of the elderly people dwell at their community and live together with their family and relatives.

***Elderly abuse***

Elderly abuse has serious consequences for the health and wellbeing of older people and can be of various forms: physical, verbal, psychological/ emotional, sexual, and financial (World Health Organization 2008). In the Philippines, there seems to be no adequate laws or policies that will protect elderly people from institutional, community and domestic abuse, and violence during these fast changing times (Junio 2014). Many cases of elderly abuse are either not properly reported or not reported at all to authorities (Carlos 1999). Most elders are afraid to come out or just hide in silence because the perpetrators itself are within their family circle. They are afraid that their privacy will be lost, will suffer more mistreatment or be placed in nursing homes. Hence, elderly leave the abuser to God’s intervention and just pray that such predicament will eventually stop whenever they suffer in silence.

The Philippine society is also becoming increasingly youth-centric, with aging regarded as something similar to a disease (*The Philippine Star* 2016). Children live for the moment and no longer feel responsible for the care of older family members. Their refusal or failure to fulfill a caregiving obligation may inflict psychological and emotional distress on the older people. Thus, elderly could suffer neglect from members of their own family.

***Health status***

According to the Commission on Population (PopCom), Filipinos are living longer but in poor health. This demographic shift entails various elderly-related issues like rehabilitation, depression, functional ability, and health care. Moreover, the pattern of disease at the end of life is changing and elderly people suffer from the double burden of degenerative and communicable diseases (Philippine Country Report 2007 & Department of Health 2005). Noncommunicable diseases such as cardiovascular diseases and cancer are the leading causes of mortality for this age group. The leading causes of morbidity, on the other hand, are infectious in nature, such as influenza, pneumonia, and tuberculosis. Other common health-related problems among older persons are difficulty in walking and chewing, hearing and visual impairment, osteoporosis, arthritis, and incontinence (*Philippine Statistics Authority* 2005, Reyala 2000, Department of Health 2005). Disabilities and impairment in function increase with age and adversely affect the quality of life of older persons.

Mental illness is the third most common form of disability after visual and hearing impairments as reported in the disability survey conducted by the National Statistics Office in 2000. There is an average of 88 reported cases of mental illness per 100,000 Filipinos (*Philippine Statistics Authority* 2013). In a separate study by the Social Weather stations, it was said that 0.7% of the total household in the Philippines in 2004, have a member with mental disability (Department of Health 2005). The most reported cases of mental illness are often linked with familial or hereditary mental disorders including schizophrenia alongside with mental illness linked with psychosocial development. According to the Baseline Survey for the National Objectives for Health (BSNOH) conducted in 2000, the more frequently reported signs and symptoms of mental health problem were excessive sadness, confusion, forgetfulness, delusions and no control over the use of cigarettes and alcohol (Department of Health 2016, *The Manila Times* 2014). Excessive sadness, forgetfulness, confusion and delusions, increase with age while cigarette and alcohol abuse affect adults and adolescents more than the older persons. The prevalence of mental illness, since it can be chronic, is reported to be highest among the older age groups.

In the Philippines, public awareness on mental health problems are hampered by scarce budget, lack of enabling law and dedicated manpower (*The Manila Times* 2014). While a national mental health policy was signed in 2001, no mental health legislation was done for the policy to have a legal framework. Thus, a mental health act is recommended for endorsement.

Mental health services and programs should not be an isolated effort. It must be integrated to cascade them to the community level. The social stigma associated with mental illness is a major cause for non-use of health and psychosocial services. The lack of understanding of mental illness and the importance of mental health is as serious as the lack of a regular and useful database on the prevalence, manifestations, causation, and risk factors of mental illness in the country.

***Ageism***

Ageism can most simply be defined as negative attitudes or behaviors toward an individual solely based on that person’s age (Nelson 2004). Age discrimination in the Philippines has become such a pressing issue that not only affects the labor force but most importantly, the personal and emotional wellbeing of the elderly (Word Press 2012). Elderly may suffer from lower self-esteem, and feelings of stress and anxiety (Orenstein 2012). For instance, senior workers over 50 may feel that their worth in the work force is no longer important and become an easily replaceable member. Those who are forced to retire and still physically able must search for another source of income in order to live more comfortably.

***Quality of life***

Quality of life is a broad ranging concept affected in a complex way by the person’s physical health, psychological state, level of independence, social relationships, personal beliefs and their relationship to salient features of their environment (World Health Organization n.d.). For the elderly people, the ability to engage in daily activities, independence and functional state is their definition of health-related quality of life (Cleary & Howell 2006).

The recent worldwide recession aggravated by the lack of safety nets and social protection remains to be the primary obstacle to achieve quality of life especially among elderly people (Sanchez 2008). It is the most substantial issue that is being faced by every country, most especially developing countries like the Philippines in which the average poverty incidence of population is 26.3% (*Philippine Statistics Authority* 2016). At present, the heavier weight of the responsibility of caring for the elderly is on the shoulders of Filipino families, not on the government (De Leon 2014). The pension system benefits only those who have been employed in formal and regular work which is something that only few people enjoy. The majority, especially the marginalized groups of farmers, fishermen, laborers, and informal workers, rely on the care of their family members and relatives for their wellbeing in old age. Unfortunately, even this traditional kind of support is gradually deteriorating which is triggered mainly by family’s economic and social instability.

Aside from the psychological or emotional aspect of getting older, many elderly people are worried of losing employment opportunities (De Guzman 2011). The government is not providing job opportunities for those elderly who are still competent and physically able. Thus, their dependence on their children is due to lack of personal financial capabilities. Poor government supported health programs further exacerbate the situation. The limited range of public geriatric services alongside the rising cost of living also put much strain to Filipino families to provide for a good quality of life for their elderly members. For instance, the Philippine General Hospital has a geriatric ward with only a few doctors specializing in Gerontology and Geriatrics. There is always a long queue of elderly people wanting to avail of free consultation and medication but not all are accommodated due to lack of supplies and limited doctors. Medical and hospital costs often consume a significant proportion of the savings of the elderly people. And even if they are granted by a 20% discount on medical and dental services, it is still tough to get sick. Nevertheless, just recently, the government approved mandatory Philippine Health (PhilHealth) insurance coverage for all senior citizens regardless of their social and economic status (Subillaga 2015). This will somehow ease the tension of the compelling health care issues affecting the rapidly aging population.

**Situational Analysis**

The City of Muntinlupa is the southernmost city in the National Capital Region. It is a highly urbanized city and 19th most populous area in the country. The city has a population (2015) of 504,509 in which 5.63% are senior citizens. It has a total 28,403 senior citizens, 43.8% are males and 56.2% are females. Muntinlupa has two legislative districts. District I consists of 4 barangays (Bayanan, Putatan, Poblacion and Tunasan) whereas District II consists of 5 barangays (Alabang, Ayala-Alabang, Cupang, Buli and Sucat).

**Problem Analysis**

Preliminary meeting with the City Health Officer (CHO), Office of the Senior Citizens Affairs (OSCA) Officer in Charge and the City Mayor was held last August 2016 to collaborate and discuss problems in the community. Key informant interviews with selected members of the barangay hall and health centers were also held to know more about the community. Based on the preliminary investigation and criteria for problem prioritization, mental health seems to be the most urgent and timely health problem affecting the senior citizens in the target area. Henceforth, the project ENGAGE was conceptualized which stands for Embracing and Nurturing Global AGEing.

**Criteria for Problem Prioritization**

**
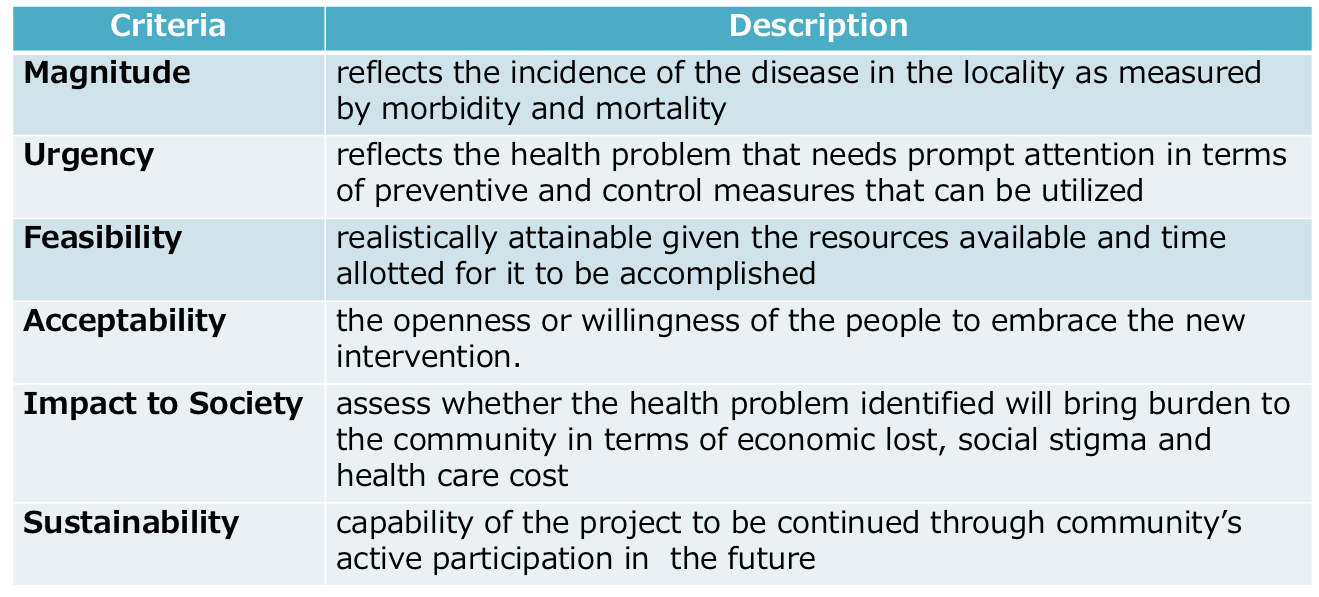
**

**Decision matrix for problem prioritization**

**
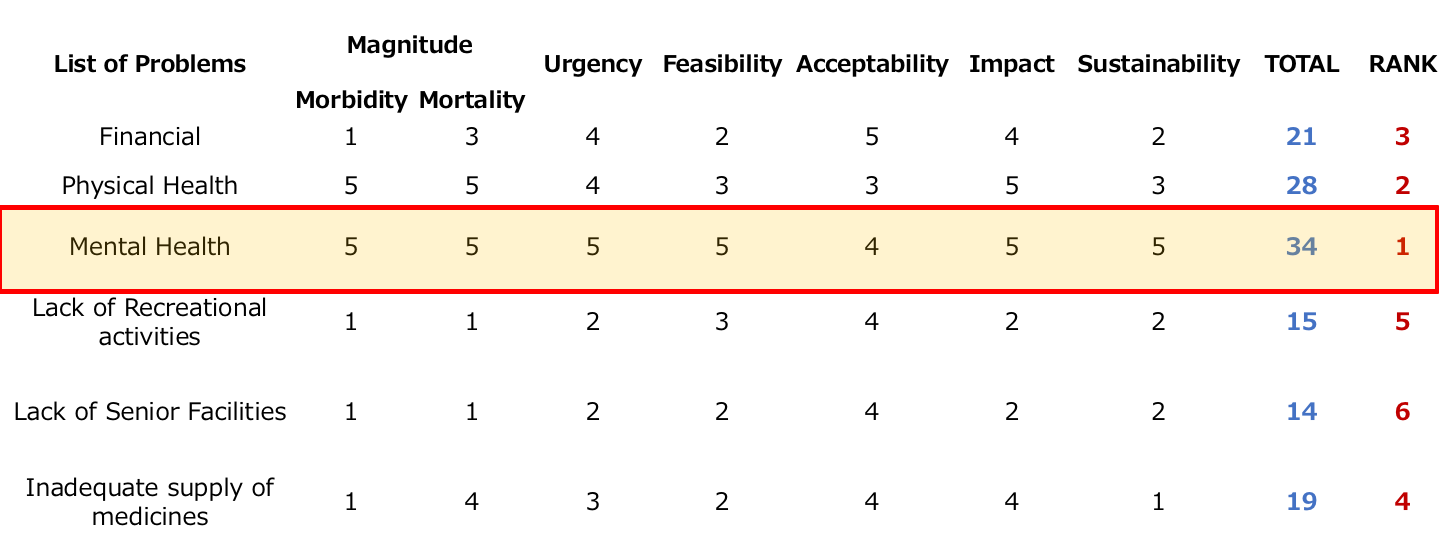
**

**Aging and Mental Health**

Mental or neurological disorder affects over 20% of adults aged 60 and above. It also accounts for 6.6% of all disability in this age group (WHO 2016). The most common neuropsychiatric disorders among older people are dementia and depression. Dementia is a syndrome in which there is deterioration in memory, thinking, behavior and the ability to perform everyday activities. It mainly affects older people, although it is not a normal part of ageing. Depression, on the other hand, can cause great suffering and leads to impaired functioning in daily life (WHO 2016). Symptoms of depression are often overlooked and untreated because they coincide with other problems encountered by older adults. Hence, depression is prevalent but underdiagnosed and undertreated in primary care settings.

In the Philippines, a survey conducted in the province of Rizal showed a 6.6% rate of depression (Oro-Josef, 2014). This prevalence rate among the elderly shows that depression can be present in healthy Filipino communities. It is also considerable to note that a fourth (26.5%) of the population has scores suggestive of depression. This is a window for an early intervention in the community level.

Screening the elderly population for possible depression is important to reduce health care utilization and improve wellbeing for this age group. It is necessary to identify older people with scores suggestive of depression to avoid development of outright depression.

**PROJECT DESCRIPTION**

***Rationale***

Mental health problems commonly arise in the older population, but frequently remain undetected and untreated (Manaf 2016). These problems are caused by the complex interaction of social, psychological and biological factors (WHO 2016). Recently, Filipinos’ mental illness has been increasing and it affects around 10-15% of children and 17-20% of adults (Department of Health 2016). Their major symptoms include excessive sadness, delusion, confusion, and forgetfulness. Additionally, more Filipino senior citizens are committing suicide due to depression (Pilapil 2017). This is associated with their inability to adapt to the rapid social and economic developments (Carlos 2016). Hence, emotional stability and wellbeing are not connected with increasing age.

In spite of this growing concern, mental health has been overlooked in the Philippines. For example, legislation is not available which can provide a legal framework to all endeavors involving mental health. This is further complicated by the lack of dedicated manpower and facilities and the shortage of human resources (Department of Health 2012). The Department of Mental Health and Substance Abuse (2011) documented that only about 700 psychiatrists and approximately 1000 psychiatric nurses can address the mental health-related issues in the country.

To date, little is known about the subjective psychological wellbeing of senior citizens in the Philippines. Moreover, the prevalence of geriatric depression and its associated risk factors have not yet been explored. Also, differences in sociocultural environment between Asia and Western countries may reveal different dynamics of these associated risk factors. Therefore, more research efforts are required to clarify this relationship.

The **Project ENGAGE** (Embracing and Nurturing Global Ageing) aims to decrease the prevalence of depression among community-dwelling seniors and will be initiated as an action research project in the City of Muntinlupa, Philippines. This research project will first identify the determinants of depression among community-dwelling seniors. It will then promote peer counseling program and community-based mental health activities. In the end, it can improve the psychological wellbeing of seniors most especially those who are suffering from depression.

**PHASE 1: DETERMINANTS OF DEPRESSION AMONG**

**COMMUNITY-DWELLING SENIORS**

**OBJECTIVES**

1. To examine the socio-demographic and psychosocial risk factors of depression among senior citizens in the City of Muntinlupa
2. To detect the subjective psychological wellbeing and depression among the elderly population
3. To determine the association between depression and socio-demographic and psychosocial risk factors among the elderly

**CONCEPTUAL FRAMEWORK**


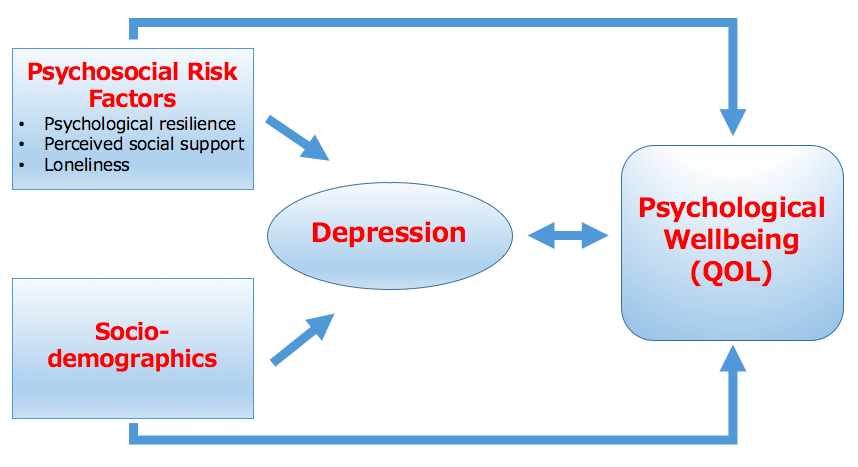


Figure 1. Conceptual Framework on the Determinants of Depression among

Community- dwelling Seniors

Socio-demographics and psychosocial risk factors are the two independent variables that may affect the onset of depression among older people. These two variables may also affect the psychological wellbeing of this age group. The outcome variables in this study are depression and psychological wellbeing. The two may have a reversible relationship.

**METHODS**

**Study design**

The study design will be a community-based, **cross-sectional study**.

**Study area**

The study will be conducted in Muntinlupa City which is the southernmost city in the National Capital Region, the most populated region in the Philippines. Muntinlupa is classified as highly urbanized city with a poverty incidence of 1.9% in 2012 (Philippine Laws, Statutes & Codes 1995, *Philippine Statistics Authority* 2016). The city has one of the highest records of senior citizens, which account for 5.63% of its population (Socio-economic profile n.d.).

**Participants and selection criteria**

Participants of this study are community-dwelling seniors in the City of Muntinlupa. The city has nine barangays and we will include all barangays in our data collection. Ayala-Alabang, though it is difficult to access, will do the recruitment through church associations, citizen meetings or professional groups.

Those aged 60 years old and above are regarded as senior citizens in the Philippines (Ericta 2012). Therefore, we will select Filipino senior citizens who are registered members of the Office of Senior Citizen Affairs (OSCA) randomly from the seniors’ registry of the OSCA. We will only ask for the name, address and contact information (if there is any) of the senior citizens. The OSCA list will be encrypted with a password and document sharing is forbidden. Only the primary investigator will have access on that list. This is in response to the Data Privacy Act of 2012. If the list cannot be obtained, maximum variation sampling will be done based on the percentage of seniors per barangay. Participants will then be recruited purposively by the primary investigator and trained BHWs through home visits. Each BHW has their own list of senior citizens in their catchment area. We will use the list for house to house recruitment. The primary investigator and BHWs will invite the senior citizens to participate in the study. All participation by seniors shall be voluntary, and participants must give their informed consent prior to participation.

Participants of the Phase 1 must possess a valid senior citizen’s identification card to be eligible. We will exclude those elderly people in long-term care, with terminal diseases, or with moderate/ severe cognitive impairment and currently suffering from deafness, aphasia or other communication disorders.

For the sampling procedure, we used probability proportionate to size techniques. We calculated the sample size using Open Epi version 3.01 with the following set parameters: population size of 504,509, anticipated % frequency of 5.63, absolute precision of 2%, design effect of 1, with 95% confidence level, and assuming a 10% dropout rate. Then we will invite at least 561 community-dwelling seniors for a face-to-face interview to answer a standardized questionnaire. We will train a number of Barangay Health Workers from each barangay on how to administer the survey questionnaire. We will first prepare in English those scales which do not have a Filipino version and translate them later to the Filipino language. We will modify the questionnaire (a) after comments of the experts and sampled senior citizens, and (b) after pretesting to twenty-five randomly selected senior citizens and will be altered accordingly. We will then back translate the questionnaire to English for data analysis with the guidance of independent local researchers.

**Data collection and study tools**

We will check the validity and reliability of the scales in the Philippines prior to the study. We will do face validity by asking experts to evaluate the scales for clarity, appropriateness for the Philippine context, and ability to assess the topic under investigation. We will also do content validity and reliability test through pretesting of the questionnaire to twenty-five randomly selected senior citizens. We will ask the comments of the sampled senior citizens and modify the questionnaire accordingly. We will then measure the reliability of the scales by Cronbach’s alpha.

**Outcome variable**

1. Depression
2. Subjective Psychological Wellbeing

We will measure the **depression** status of the senior citizens by the 15-item Geriatric Depression Scale (GDS). It is specifically developed for use in geriatric patients and contains fewer somatic items (Yesavage 1983; Sharp & Lipsky 2002). A score of 5 or more is suggestive of depression.

We will measure **subjective psychological wellbeing** using the 5-item WHO Wellbeing Index (WHO-5). The scale has adequate validity both as a screening tool for depression and as an outcome measure in a wide range of study fields (Topp 2015). The raw score is calculated by totaling the figures of the five answers. The raw score ranges from 0 to 25, 0 representing worst possible and 25 representing best possible quality of life. In order to monitor possible changes in wellbeing, we will use the percentage score. A 10% difference indicates a significant change.

**Independent variables**

1. Socio-demographics
2. Psychosocial risk factors

- Psychological resilience

- Perceived social support

- Loneliness

**Socio-demographics**

This survey will include socio-demographic information such as age (continuous), gender, marital status (married/ remarried, never married, divorced/separated, widowed), education level (no education, primary, secondary, tertiary), occupation, monthly income (no income, poor, average, good) and pension (no pension, government pension, private pension, social pension).

We will identify the health status by the number of chronic diseases and self-rated health status. The list of chronic diseases includes hypertension, coronary disease, cerebrovascular disease, diabetes, chronic bronchitis, spondylosis or osteoarthritis, pulmonary disease (asthma, emphysema), cancer, and others (if not in the list). We will measure self-rated health status on a 5-point scale ranging from very good to very bad.

We will also include the living arrangement (living alone, living with only spouse, living with only children, living with spouse and children) and vices such as smoking (never-smokers, ex-smokers, current smokers) and alcohol intake (nondrinkers, occasional drinkers, daily drinkers) in this survey.

**Psychosocial risk factors**

We will measure the **psychological resilience** of the seniors by the Resilience Appraisal Scale (RAS) which contains 12 questions through which seniors indicate the degree of applicability of each statement to them using a five-point Likert scale (“strongly disagree” to “strongly agree”). The RAS consists of three parts of coping skills which evaluate perceived abilities in social support seeking, emotional regulation, and problem solving (Johnson et al. 2010). The total RAS score ranges from 12 to 60, with a higher score indicating higher perceived psychological resilience.

We will assess **perceived social support** using the 10-item Duke Social Support Index (DSSI), which was developed to measure two important constructs related to social support such as social satisfaction and social interaction (Landerman, et al.1989 & Wardian et al. 2012). The possible score ranges from 10 to 30. Higher scores indicate a higher level of perceived social support among participants.

We will measure **loneliness** by the 8-item UCLA Loneliness Scale (ULS-8). The scale employs a 4-point Likert scale with values ranging from “never” to “always” (Hays & DiMatteo 1987) and the total score ranges from 8 to 32. There was no cut-off score identified to define loneliness. However, a higher score on this scale indicates more intense feelings of loneliness.

**Data analysis**

The principal researcher anonymized all completed questionnaires with individual numbers. We will do computer and manual checking to review inconsistent data and remove outliers. Incomplete questionnaires will be omitted from the final analysis.

The principal researcher will enter the data using Epidata Version 3.1. We will use descriptive statistics to summarize the data and cross tabulation to identify their distribution. We will employ correlation statistics to investigate the relationship between each study variable. After that, we will perform hierarchical regression analysis (Cohen, West & Aiken 2003) to ascertain the predictors of depression among the participants. We will set the level of significance to 0.05 (two-tailed), and perform statistical analyses using Stata 13.1 (StataCorp, College Station, TX, USA).

**PHASE 2 – TRAINING SENIOR VOLUNTEERS ON LEADERSHIP AND PEER**

**COUNSELING**

**OBJECTIVE**

To evaluate the effectiveness of the training programs on improving senior volunteers’ knowledge, skills and attitudes toward counseling and facilitation of community-based activities

**CONCEPTUAL FRAMEWORK**

**
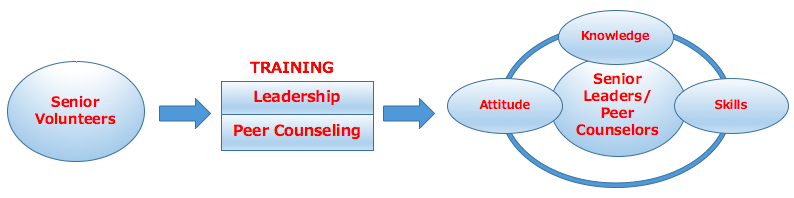
**

Figure 2. Conceptual Framework on Training Senior Volunteers on Leadership and Peer Counseling

Senior volunteers will be trained for both leadership and peer counseling for two months. The training will focus on knowledge acquisition, skill development and change in attitude. It is hope that by the end of the training, senior volunteers will become senior leaders / peer counselors.

**METHODS**

**Study design**

The study design will be a **pre- and post-intervention study**.

**Study area**

The same as above

**Participants and selection criteria**

We will recruit a total of 60 senior volunteers from the eight barangays. We will have 7-8 senior volunteers per barangay. In this pilot study, we did not perform any sample size calculation. Based on the literature and the type of data analysis we will do (Wilcoxon signed-ranked test and thematic analysis), at least 30 subjects are enough to give reliable results (Burmeister 2012; Akeyede, et al., 2014). Meanwhile, in a previous study conducted by Joo et al., they only recruited 6 peer mentors to meet 30 patients. Also, the number of participants for this study was decided on the basis of location/ space, manageability and financial resources. Thus, there will be two batches for the training. Each batch consists of 30 senior volunteers.

Inclusion criteria: They must be 60 years old and above and are registered members of the Office of the Senior Citizens Affairs. They must be free from depression based on the screening result provided by the Geriatric Depression Scale (GDS) score. Their GDS score must be 4 or below.

Exclusion criteria: Those who already participated in the Phase 1 of this study and who got a score suggestive of depression will be excluded.

**Data collection and study tools**

We will create a pre-structured training design for both peer counseling and leadership training. We will conduct training needs assessment through a pre-test to identify which topic needs more emphasis during the training and what concepts have common misconceptions. We will gather training expectations from the senior volunteers. We will ask them about their expectations for the content of the training, methodology, and expectations from the resource persons and fellow participants.

We will also design a semi-structured questionnaire to be administered by the principal researcher. We will use this questionnaire to explore the impact of the training programs on the senior volunteers’ attitudes towards leadership and peer counseling. This will be carried out by face-to-face interviews before and after the training.

We will then do lecture discussions to learn new knowledge. We will use pre- and post-test scores to compare improvement of knowledge. We will evaluate the competencies expected from the senior volunteers through role-plays, community-based project proposal and field work.

Finally, we will assess the impact of the two-month training programs on the senior volunteers’ attitudes through FGDs consisting of five members per group. We will conduct two FGDs per barangay accounting for a total of 16 FGDs. We listed the questions as interview guide to explore the senior volunteers’ experience and personal growth after the training. We will then encourage them to express their views and opinions without confining to the questions being asked.

**Data analysis**

We will use Wilcoxon signed-ranked test to compare the changes in knowledge on pre- and post-intervention data. We will assess peer counseling competency through role-plays and fieldwork using some evaluation criteria. We will evaluate their leadership skill through a project proposal which is the practical application of what they have learned from the training. We set an average rating of at least 70% to determine whether competencies asked to be portrayed are achieved. For semi-structured interviews, we will transcribe the recorded notes verbatim and translated into English. We will analyze with the aid of NVivo using combinations of inductive and analytical approaches (Hammond 2004). Five researchers will be involved in this process and every transcribed interview will be analyzed by at least two researchers to test the reliability of the interpretations. We will present the findings as themes that emerged from the analysis of 16 transcripts.

**PHASE 3 – ATTENUATION OF GERIATRIC DEPRESSION AMONG**

**COMMUNITY-DWELLING SENIORS**

**OBJECTIVE**

To evaluate the effectiveness of the interventions on the level of depression and psychological wellbeing of community-dwelling seniors.

**CONCEPTUAL FRAMEWORK**

**
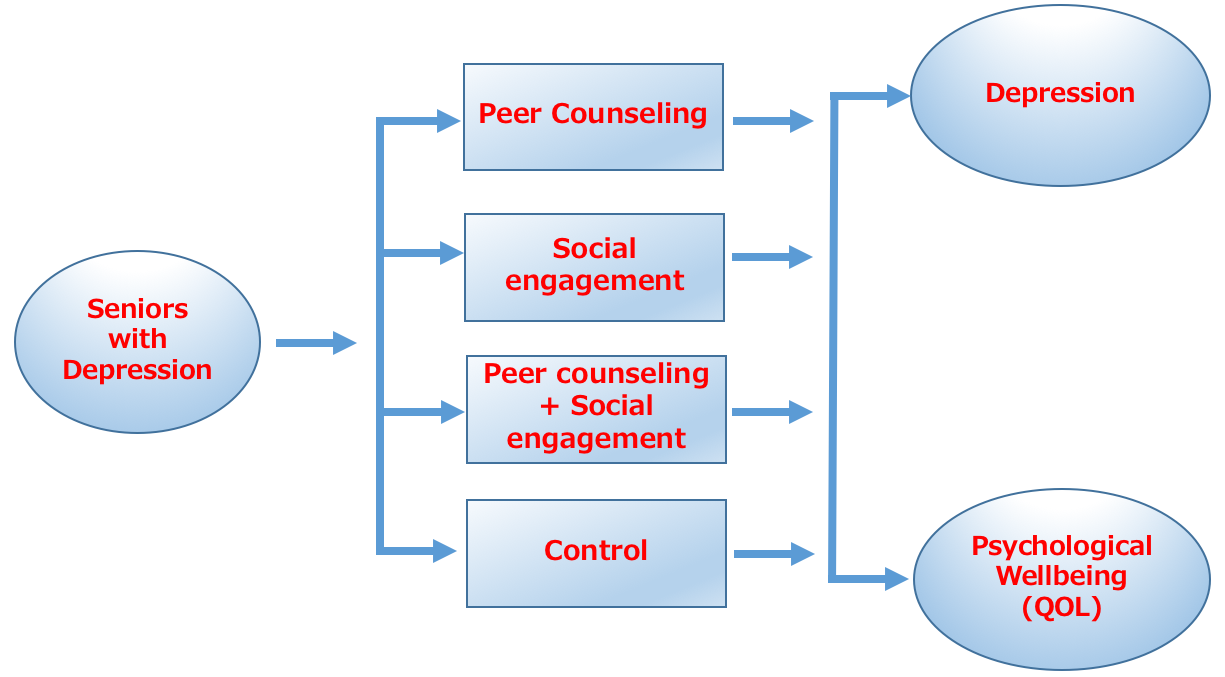
**

Figure 3. Conceptual Framework on Attenuation of Geriatric Depression among Seniors

Senior citizens who got a score suggestive of depression (GDS score of 5 and above) will be allocated into four groups: (1) peer counseling, (2) social engagement, (3) combination of peer counseling and social engagement, and (4) control. Three months after the intervention, their level of depression and psychological wellbeing will be measured to assess the impact of the interventions.

**METHODS**

**Study design**

The study design will be a **quasi-experimental study**. We will divide the seniors who got a score suggestive of depression into four groups. The first group (n = 60) will only receive peer counseling; the second group (n = 60) will join community activities; the third group (n = 60) will join both social engagement and peer counseling, and the last group (n = 60) will neither take part in any activities and will serve as the control group. We will analyze all the participants and assess the impact of the inventions after three months.

**Study area**

The same as above

**Participants and selection criteria**

We will include senior citizens who got a score suggestive of depression (GDS score of 5 and above) in this study. We will allocate them purposively into four groups: (1) peer counseling, (2) social engagement, (3) combination of peer counseling and social engagement, and (4) control. We used Open Epi version 3.01 and based the following parameters from a meta-analysis (Cuijpers, 1998) of the effects of outreach programs to depressed senior citizens in the community: effect size of 0.77, power of 90%, alpha set at 0.05 (two-sided) and expected dropout rate of 25%. We calculated at least 40 senior citizens per group. Considering the small sample size, we decided to increase the sample size to at least 60 senior citizens per group.

Each senior peer counselor will be in charge of two patients. One patient will be assigned to group A (peer counseling) and another patient will be assigned to group C (social engagement + peer counseling). The number of participants for social engagement (n = 60) is decided on the basis of location/ space, manageability and financial resources. There will be two batches for the social engagement group. Each batch consists of 30 participants.

**
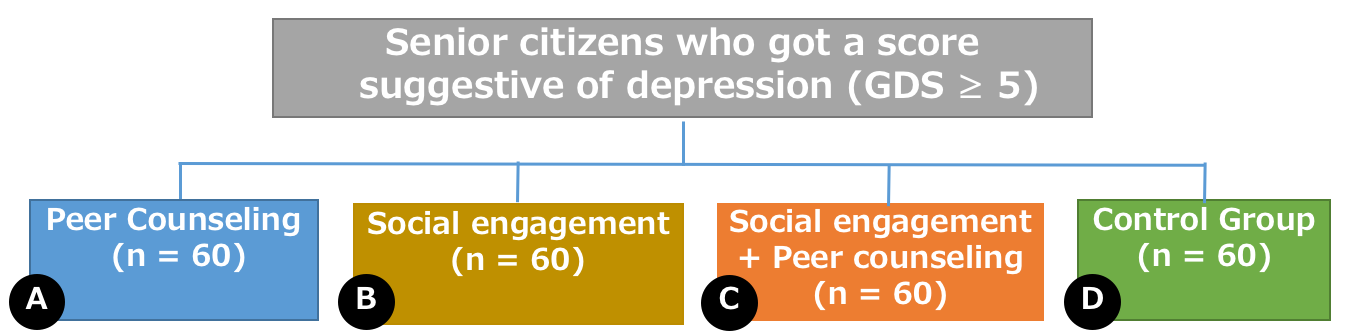
**

Figure 4. Participants and selection criteria for Phase 3

**Data collection and study tools**

**Peer Counselor – Patient Meetings** - Peer counselors will do home visits to their assigned patients for 1-hour every week for three months. The goals of the meetings are to establish a strong working alliance, identify patient-defined problem, encourage behavior change, and facilitate engagement with the community. At the initial visit, the peer counselor will ask the patient what they would like to get out of the meetings in order to establish a patient-identified goal that they can work on together. Peer counselors will accomplish weekly reports for documentation purposes. Both the peer counselors and patients will complete the Working Alliance Inventory Short Form (WAI-SF) pre- and post-intervention (Joo 2016). Patients will also answer the same set of questionnaires for psychological wellbeing, depression and psychosocial risk factors after study completion. Peer counselors and patients will also be interviewed to assess their experiences of the intervention. The principal researcher will conduct in-depth interviews with each patient and FGDs with the peer counselors (5 members per group) using a semi-structured questionnaire.

**Peer Counselor-Psychologist Supervision Meetings** – The psychologists will meet with the peer counselors once a month for an hour for supervision and collaboration. During meetings, the peer counselors will report on patient’s progress and shared impression and insights. The mental health professional will then provide guidance, reinforcement and constructive feedback to continue skills development of the peer counselors.

**Community-based Mental Health Activities** – We will implement the project proposals presented during the training in the community. The trained senior volunteers and health providers will facilitate the activities weekly for three months. We will then collect the same set of data for psychological wellbeing, depression and psychosocial risk factors after the intervention. We will also conduct FGDs consisting of five members per group for both senior participants and trained senior volunteers. We will hold eight FGDs for senior participants and 16 FGDs for trained senior volunteers. We listed the questions as interview guide to explore the trained senior volunteers’ and senior participants’ experience and personal growth after the intervention. We will explore their acceptability and motivation to continue the activities. We will encourage them to express their views and opinions without confining to the questions being asked.

**Data analysis**

We will carry out pre- and post-intervention comparisons followed by analysis of semi-structured interview data. We will use Cochran-Mantel-Haenszel method to test for statistical significance of the correlation between each study variable. We will set the level of significance to 0.05 (two-tailed), and perform statistical analyses using Stata 13.1 (StataCorp, College Station, TX, USA). For semi-structured interviews, we will transcribe the recorded notes verbatim and translated into English. We will analyze with the aid of NVivo using combinations of inductive and analytical approaches (Hammond 2004). Five researchers will be involved in this process and every transcribed interview will be analyzed by at least two researchers to test the reliability of the interpretations. We will present the findings as themes that emerged from the analysis of 176 transcripts for the peer counseling and 24 transcripts for the community-based activities.

**PHASE 4 –SUSTAINABILITY OF ENGAGE PROJECT**

The last component aims to ensure the sustainability of the action research project through a legislative support from the policy makers particularly from the City Council on Health. We hope to generate the support for the adoption of the implementation of Peer Counseling Program and Community-based Mental Health Activities in the municipality. We will conduct an interview to relevant stakeholders who have witnessed our activities in the said program. By doing so, we can strengthen policy implications. We also would like to create Task Force ENGAGE that will serve as the vehicle for consolidation and unification efforts on Aging and Mental Health. The framework will hopefully be integrated in city ordinances. The draft ordinances are expected to be appraised and be approved by the City Council. The development of these policies will be coordinated with the Local Health Board in partnership with the Office of Senior Citizen Affairs and Barangay officials. We aim to draft a Memorandum of Agreement to be signed during the disengagement ceremony.


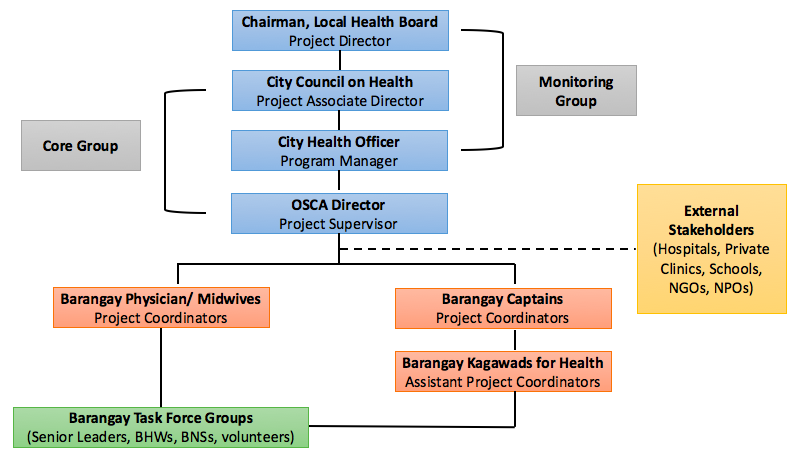


Figure 5. Organizational Chart of Project ENGAGE

The monitoring group will be the LHB and CHO whereas the OSCA and City council on Health will be the core group. All the senior citizen programs will be cascaded down to the barangay level in coordination with the Barangay captain and Barangay physician/ midwives. The barangay task force groups will consist of the senior leaders, BHWs, and other volunteers. Relevant stakeholders can coordinate with the OSCA director for partnership.

**STUDY IMPLICATIONS**

The identification of risk factors of psychological wellbeing and depression in late life, especially the psychosocial factors, is key to improve the quality of life of community-dwelling seniors. The active involvement of seniors in community activities and peer counseling will improve public awareness about mental health and reduce the stigma associated with aging and mental illness. The senior volunteers, once empowered and trained properly, could serve as high potential human resources to improve the psychological wellbeing of their peers. The success of the pilot study can be used as a framework to scale up future mental health interventions.

**PROJECT TIMELINE**

**
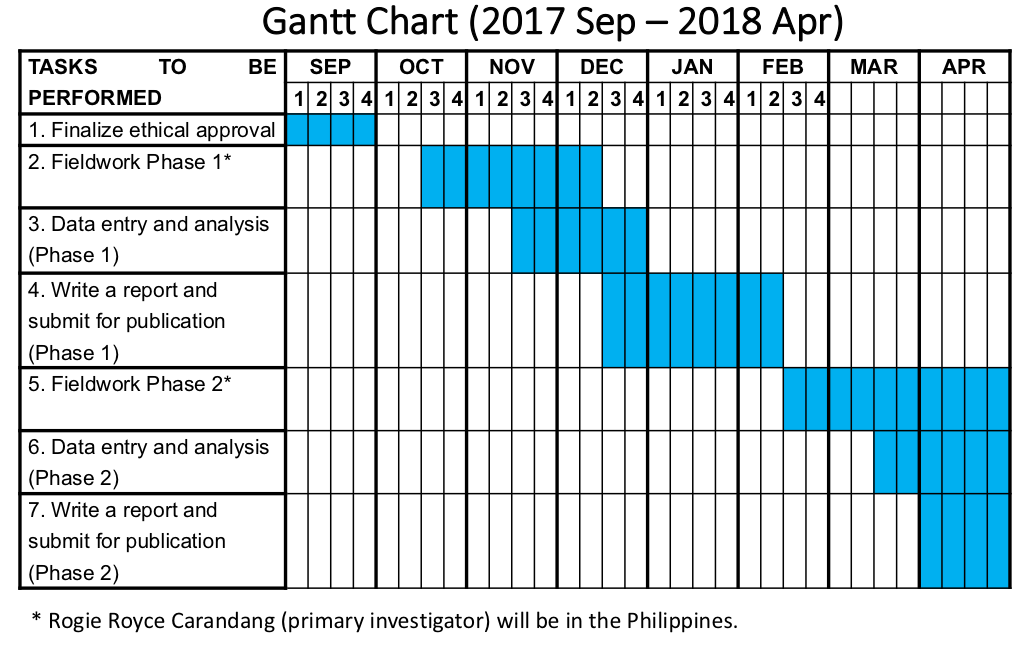
**

**
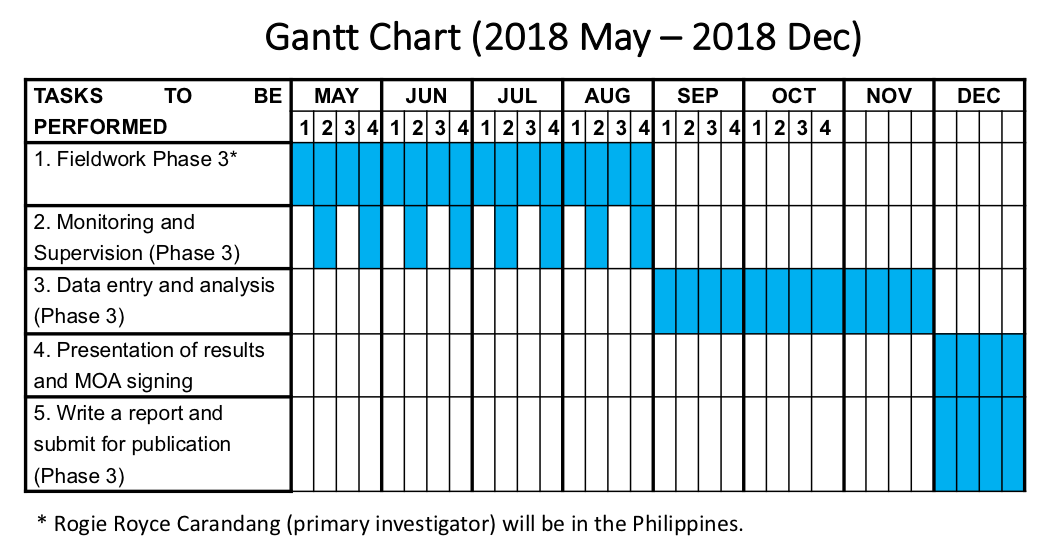
**

Phase 1 (two months): 3^rd^ week of October – 2^nd^ week of December 2017

Phase 2 (two and a half months): 3^rd^ week of February – 4^th^ week of April 2018

Phase 3 (4 months): 1^st^ week of May – 4^th^ week of August 2018

* The primary investigator will be present in all phases of the study

**ETHICAL CONSIDERATIONS**

Ethical approval will be sought from the Research Ethics Committee of the University of Tokyo and the University of the Philippines Research Ethics Board. We will obtain the permission of community dwelling seniors by giving a letter of consent/permission to conduct research involving them. All participation by seniors shall be voluntary, and participants must give their informed consent prior to participation. In addition, participants must be allowed to withdraw from the study at any time without penalty, and they have the right to obtain the results of the study if they so wish. We will be aware not to cause emotional harm to the participants by being careful and sensitive during interview and training sessions. Personal biases and opinions shall not get in the way of the research.

The following are the salient points to ensure the safety of the study participants:

**Withdrawal criteria**

**Phase 1**

All participation is voluntary. A participant can leave Phase 1 of the study at any time (during or 6 months after answering the survey questionnaire). When withdrawing from the study, participants should let the research team know that he/she wishes to withdraw. They can either sign the withdrawal form and hand it to the researcher or contact the researcher from the information written in the information sheet.

A participant may provide the research team with the reason(s) for leaving the study, but is not required to provide his reason. If a participant decided to withdraw, all the services he receives from the OSCA will continue and nothing will change.

**Phase 2**

A participant does not have to take part in the Phase 2 of the study if he does not wish to do so. All participation is voluntary. If he decides to participate, he is free to change his mind and stop being in the study at any time (during or 6 months after the last interview) without any harm. They can either sign the withdrawal form and hand it to the researcher or contact the researcher from the information written in the information sheet. If a participant decided to withdraw, all the services he receives from the OSCA will continue and nothing will change.

A participant may be asked to withdraw as a senior leader/ peer counselor if he cannot accomplish his obligations such as:

- Holding a weekly home visit to his clients;
- Submiiting progress reports of his clients;
- Facilitating the assigned community activities; and
- Receiving positive feedback from the client and from his/ her family members

As part of the contingency plan, the participant who withdraw from the study will be

replaced by another senior leaders / peer counselors.

**Phase 3**

A participant does not have to take part in the Phase 3 of the study if he does not wish to do so. All participation is voluntary. If he decides to participate, he is free to change his mind and stop being in the study at any time (during or 3 months after the last interview) without any harm. They can either sign the withdrawal form and hand it to the researcher or contact the researcher from the information written in the information sheet. If a participant decided to withdraw, all the services he receives from the OSCA will continue and nothing will change.

The following are selected examples of participant-related reasons for study participants (SPs) to be discontinued from the study by the investigator:

- Failure to cooperate adequately with the senior leader/ peer counselor
- Failure of SP to attend specific number of meetings e.g. attended less than 70% of the meetings
- Worsening of SP's depression based on the evaluation of health professionals
- SP experiences serious discomfort and emotional outburst during the study that requires withdrawal from the study
- SP moved to another city
- Any combination of the above
- Withdrawal of SP's informed consent
- Death of SP

The following are research-related reasons for the study to be discontinued:

- Study is terminated prematurely because of unacceptable safety concerns of the procedure being done.

**Risks**

**Potential stigmatization**

This can be addressed through increasing awareness about depression. We will educate study participants and their caregivers about the nature of depression to alleviate both public and self-stigmatization. We will clear misconceptions about mental illness specifically, depression. We will make information sheets on mental health for senior citizens for psychoeducational purposes. Just simply talking about depression reduces the stigma associated with it.

**Worsening of depression**

The senior peer counselors will be trained to be sensitive to the behavioral and mood changes of their assigned patients. They can monitor the changes from their weekly visits with them. If depressive symptoms get worse, we will refer the study participants to the nearest health center or specialty clinics to receive immediate medical attention. We will provide a list of mental health professionals in the area and give the peer counselors a copy for distribution. The patients can choose where to go if they want to. It will be voluntary unless we determine a high suicide risk based on the reports of the peer counselors. We will suggest the CHO to partner with mental health professionals for referrals.

**Discomfort or emotional outburst during the study**

It depends on what kind of outburst we are talking about here. If the participants started crying or getting angry during the activity but still looks like they can manage themselves and are merely expressing, we will just allow and validate it. We can make this as a jumping point for an in-depth discussion of what emotional issues are commonly experienced by the seniors. For more serious ones, we can invite them for a more private one-on-one or referred to the clinic for intervention. The key here is for the peer counselors to get the profile of the clients (which we will do), for the peer counselors know if the client is at risk from emotional distress during the sessions. The peer counselors will be trained to do empathic listening techniques. They will reflect everything they hear/ see from the clients and show that they are interested to understand. Basically, co-journeying and supportive counseling.

Worse comes to worst, the OSCA in Muntinlupa City is located near the barangay hall and health center, so we can seek immediate help from barangay officials and health center staff in case untoward incidents happen during the study.

**Benefits**

The direct benefits of the project to the study participants is the reduction of depressive symptoms and improvement of their psychological wellbeing. The indirect benefits would be increasing awareness about depression and improvement in their health-related quality of life.

**Referral**

We will teach the peer counselors on how to screen for depression. If there is already the presence of clinical depression, peer counselors may not be effective. So, we will be referring these patients to mental health professionals. As stated above, we will provide a list of mental health professionals in the area and give the peer counselors a copy for distribution. The patients can choose where to go if they want to. It will be voluntary unless we determine a high suicide risk based on the reports of the peer counselors. We will suggest the CHO to partner with mental health professionals for referrals.

**Remuneration**

**Phase 1**

- Research assistants (trained BHWs) will receive Php 500 for 30 senior citizens interviewed.

**Phase 2 (Senior Leaders/ Peer Counselors)**

- Fare: Reimbursement (amount will be adjusted depending on actual fare of the participant). The city has an e-jeep which enable senior citizens to access free transportation. I will coordinate with the City Government to provide the service for the participants. If e-jeep is not available, approximately Php50/ participant for every training will be provided for transportation)
- Food: Free lunch, snacks, drinks
- Allowance: Php 200/ month

**Phase 3 (Depressed Seniors in the Community Activity Group)**

- Fare: Reimbursement (amount will be adjusted depending on actual fare of the participant). The city has an e-jeep which enable senior citizens to access free transportation. I will coordinate with the City Government to provide the service for the participants and their companions. If e-jeep is not available, approximately Php50/ participant for every event will be provided for transportation)
- Food: Snacks and drinks
- Allowance: Php 100 / half-day meeting
- Remuneration for the Companion: Php 50/ half-day meeting

**Recruitment**

**Phase 1**

We will randomly select the participants in the survey from the OSCA contact list. If the list cannot be obtained, maximum variation sampling will be done and recruitment of senior citizens will be done purposively through home visits. The primary investigator and trained research assistants (BHWs) will do home visits to recruit the selected participants. Each BHW has their own list of senior citizens in their catchment area. We will use the list for house to house recruitment. The primary investigator and BHWs will invite the senior citizens to participate in the study. All participation by seniors shall be voluntary, and participants must give their informed consent prior to participation. We will recruit at least 561 senior citizens in the area based on the sampling computation.

**Phase 2**

Each barangay hall has a pool of senior volunteers working in the barangay. We will recruit them and ask for their voluntary participation to become senior leaders / peer counselors. Seven to eight senior volunteers will be recruited from each barangay, a total of 60 will be asked to participate.

**Phase 3**

Senior citizens from Phase 1 who got a score suggestive of depression (GDS score 5 and above) will be eligible to join the Phase 3 of the study. We will select participants from the eligible candidates and allocate them purposively into four groups: peer counseling (n= 60), social engagement (n=60), combination of peer counseling and social engagement (n = 60) and control (n=60). Senior peer counselors in partnership with the BHWs from Phase 1 will do home visits to recruit the participants.

**REFERENCES**

1. United Nations Department of Economic and Social Affairs 2007, *World Economic and Social Survey 2007: development in an ageing world*, viewed 14 December 2016, <http://www.un.org/en/development/desa/policy/wess/wess_archive/2007wess.pdf>.
2. World Health Organization 2012, *Good Health Adds Life to Years: global brief for world health day 2012*, viewed 14 December 2016, <http://apps.who.int/iris/

bitstream/10665/70853/1/WHO_DCO_WHD_2012.2_eng.pdf>.

1. Bloom, D 2011, ‘7 billion and counting’, *Science*, vol. 333, no. 6042, pp. 562-569.
2. Christensen, K, Doblhammer, G, Rau, R & Vaupel, J 2009, ‘Ageing populations: the challenges ahead’, *Lancet*, vol. 374, no. 9696, pp. 1196-1208.
3. Beard, JR, Officer, A, de Carvalho, IA, Sadana, R, Pot, AM, Michel, JP et al. 2016, ‘The world report on ageing and health: a policy framework for healthy ageing’, *Lancet*, vol. 387, no. 10033, pp. 2145-2154.
4. Lindmeier, C & Brunier, A 2015, ‘WHO: number of people over 60 years set to double by 2050; major societal changes required’, *Media Center*, 30 September, p.1, viewed 16 December 2016, WHO Media Center Archives database.

1. Ericta, C 2012, ‘The age and sex structure of the Philippine population: facts from the 2010 census’, *Philippine Statistics Authority*, 30 August, p.1, viewed 16 December 2016, Philippine Statistics Authority Archives database.
2. World Population Prospects 2010, *Ageing and Health: Philippines*, viewed 14 December 2016, <http://www.wpro.who.int/topics/ageing/ageing_fs_philippines.pdf >.
3. Albert, JR 2012, ‘Beyond the numbers: understanding the changes in the Philippine population’, *Philippine Statistics Authority*, 16 November, p.1, viewed 16 December 2016, Philippine Statistics Authority Archives database.
4. Abejo, S (eds) 2004, *9^th^ National Convention on Statistics, October4-5, 2004: living arrangements of the elderly in the Philippines*. National Convention on Statistics, Manila.
5. Philippine Institute for Development Studies 2012, *Filipino Elderly Living Arrangements, Work Activity, and Labor Income as Old-age Support*, viewed 17 December 2016, <http://dirp3.pids.gov.ph/ris/dps/pidsdps1231.pdf>.
6. Domingo, L & Casterline JB 1992, ‘Living arrangements of the Filipino elderly’, *Asia-Pacific Population Journal,* vol. 7, no. 3, pp. 63-88.
7. AARP Real Possibilities 2014, *Caregiving among Asian Americans and Pacific Islanders*, viewed 16 December 2016, <http://www.aarp.org/content/dam/aarp/

home-and-family/caregiving/2014-11/report_caregiving_aapis_english.pdf>.

1. Andres, T 2002, *People Empowerment by Filipino Values*, Rex Bookstore, Manila.
2. Carlos, C 1999, ‘Concerns of the elderly in the Philippines’, *Philippine Social Sciences Review*, vol. 56, nos. 1-4, pp. 1-40.
3. Virola, R 2011, ‘Statistically speaking: seniors’ moments’, *Philippine Statistics Authority*, 11 July, p.1, viewed 16 December 2016, Philippine Statistics Authority Archives database.
4. Ubalde, C 2011, ‘So old, so poor: official data count elderly among poorest sectors’, *Interaksyon*, 12 July, p. 1, viewed 18 December 2016, Interaksyon Archives database.
5. World Health Organization 2008, *A global response to elder abuse and neglect: building primary health care capacity to deal with the problem worldwide: main report*, viewed 14 December 2016, <http://apps.who.int/iris/bitstream/10665/

43869/1/978924156358_eng.pdf>.

1. Junio, L 2014, ‘Like children, elderly persons also need protection from abuse’, *Interaksyon*, 30 June, p. 1, viewed 18 December 2016, Interaksyon Archives database.
2. Anon. 2016, ‘Editorial: support for the elderly’, *The Philippine Star*, 4 October, p. 1, viewed 18 December 2016, The Philippine Star Archives database.
3. Philippine Country Report 2007, *Community services for the elderly in the Philippines: a collaboration of the Department of Social Welfare and Development and the Department of Health*, viewed 20 December 2016, <http://www.mhlw.go.jp

/bunya/kokusaigyomu/asean/asean/kokusai/siryou/dl/h19_philippines.pdf >.

1. Anon. 2005, ‘Senior citizen comprised six percent of the population’, *Philippine Statistics Authority*, 18 March, p.1, viewed 16 December 2016, Philippine Statistics Authority Archives database.
2. Reyala, J 2000, *Community health nursing services in the Philippines*, Community Health Nursing Section, National League of Philippine Government Nurses, Manila.
3. Anon. 2013, ‘Persons with disability in the Philippines: results from the 2010 census’, *Philippine Statistics Authority*, 10 January, p.1, viewed 17 December 2016, Philippine Statistics Authority Archives database.
4. Department of Health 2005, *National objectives for health: Philippines 2005-2010*, viewed 20 December 2016, <http://www.doh.gov.ph/sites/default/files/publications/

NOH2005.pdf>.

1. Department of Health 2016, *National objectives for health: Philippines 2011-2016*, viewed 20 December 2016, <http://www.doh.gov.ph/sites/default/files/publications/

noh2016.pdf>.

1. Anon. 2014, ‘Mental health problems to pose heavy burden by 2030’, *The Manila Times*, 16 March, p. 1, viewed 21 December 2016, The Manila Times Archives database.
2. Nelson, T 2004, *Ageism: stereotyping and prejudice against older persons*, The MIT Press, England.
3. Anon. 2012, *Age discrimination in the Philippines and its effects on the emotional wellbeing of the aging Filipino*, Word Press, viewed 22 December 2016, <https://

growingoldorjustaging.wordpress.com/2012/03/14/age-discrimination-in-the-philippines-and-its-effects-on-the-emotional-well-being-of-the-aging-filipino/>.

1. Orenstein, B 2012, *7 ways to overcome ageism*, Everyday Health, viewed 23 December 2016, <http://www.everydayhealth.com/longevity/emotional-wellness/fighting-age discrimination.aspx>.
2. World Health Organization 2007, *Women, ageing and health: a framework for action*, viewed 18 December 2016, <http://www.who.int/ageing/publications/

Women-ageing-health-lowres.pdf>.

1. World Health Organization n.d., *WHOQOL: measuring quality of life*, viewed 20 December 2016, <http://www.who.int/healthinfo/survey/whoqol-qualityoflife/en/>.
2. Cleary, K & Howell, D 2006, ‘Using the SF-36 to determine perceived health-related quality of life in rural Idaho seniors’, *Journal of Allied Health*, vol. 35, no. 3, pp. 156-161.
3. Sanchez, N 2008, *The Filipino senior citizen: at a glance*, Sociology of Aging, viewed 23 December 2016, < https://www.academia.edu/3539922/THE_FILIPINO

_SENIOR_CITIZEN_AT_A_GLANCE?auto=download >.

1. Anon. 2016, ‘Poverty incidence among Filipinos registered at 26.3%, as of first semester of 2015’, *Philippine Statistics Authority*, 18 March, p.1, viewed 21 December 2016, Philippine Statistics Authority Archives database.
2. De Leon, A (eds) 2014, *The quality of life of the Filipino elderly in selected cities and provinces, September 2014*. 1^st^ Multipartite regional meeting on the financial security of older women in East and Southeast Asia, Manila.
3. De Guzman, S 2011, ‘Respect the elderly’, *The Philippine Star*, 30 May, p. 1, viewed 22 December 2016, The Philippine Star Archives database.
4. Subillaga, F 2015, *Health insurance coverage at 60? Definitely,* Philhealth, viewed 22 December 2016, < https://library.westernsydney.edu.au/main/sites/default/

files/pdf/cite_Harvard.pdf>.

1. World Health Organization 2015, *World report on ageing and health*, viewed 24 December 2016, <http://apps.who.int/iris/bitstream/10665/186463/1/9789240694811_eng.pdf?ua=1>.
2. Anon. 2016, ‘Highlights of the Philippine Population 2015 Census of Population’, *Philippine Statistics Authority*, 19 May, p.1, viewed 27 December 2016, Philippine Statistics Authority Archives database.
3. Philippine Laws, Statutes & Codes 1995, *Republic Act No. 7926 – an act converting the municipality of Muntinlupa into a highly urbanized city to be known as the city of Muntinlupa*, viewed 27 December 2016, <http://www.chanrobles.com/

republicacts/republicactno7926.html#. WKQGWBJ96u4>.

1. Anon. 2016, ‘Poverty incidence among Filipinos registered at 21.6% in 2015’, *Philippine Statistics Authority*, 27 October, p.1, viewed 27 December 2016, Philippine Statistics Authority Archives database.
2. Anon. n.d., *Socio-economic profile*, Muntinlupa City’s Official Website, viewed 27 December 2016, <http://www.muntinlupacity.gov.ph/v2/>.

44. Manaf, M, Mustafa, M, Rahman, M, Yusof, K & Aziz, N 2016, ‘Factors influencing the

prevalence of mental health problems among Malay elderly residing in a rural community:

a cross-sectional study’, *PLoS ONE*, vol. 11, no. 6, pp. 1-12.

45. World Health Organization 2016, *Mental Health and older adults*,

viewed 12 April 2017, < http://www.who.int/mediacentre/factsheets/fs381/en/>.

46. Pilapil, J 2017, ‘More senior citizens committing suicide - study’, *The Manila Times*, 12

April, p.1

47. World Health Organization & Department of Health 2012, *Health service delivery profile*,

viewed 12 April 2017 <http://www.wpro.who.int/health_services/service_delivery_profile

_philippines.pdf? ua=1>.

48. Philippine Laws, Statutes & Codes 1995, *Republic Act No. 7926 – an act converting the*

*municipality of Muntinlupa into a highly urbanized city to be known as the city of*

*Muntinlupa*, viewed 27 December 2016, <http://www.chanrobles.com/republicacts/

republicactno7926.html#. WKQGWBJ96u4>.

49. Anon. 2016, ‘Poverty incidence among Filipinos registered at 21.6% in 2015’, *Philippine*

*Statistics Authority*, 27 October, p.1, viewed 27 December 2016, Philippine Statistics

Authority Archives database.

50. Topp, C, Ostergaard, S, Sondergaard, S & Bech, P 2016, ‘The WHO-5 well-being

index’, *Psychotherapy and Psychosomatics*, vol. 84, no. 3, pp. 167-176.

51. Yesavage, JA, Brink, T, Rose, T, Lum, O, Huang, V, Adey, M & Leirer, VO 1983,

‘Development and validation of a geriatric depression screening scale: a preliminary

report’, *Journal of Psychiatric Research*, vol. 17, no. 1, pp. 37-49.

52. Sharp, L & Lipsky M 2002, ‘Screening for depression across the lifespan: a review of

measures for use in primary care settings’, *American Family Physician*, vol. 66, no. 6, pp.

1001-1008.

53. Johnson, J, Gooding, PA, Wood, AM, Taylor, P, Pratt, D & Tarrier, N 2010, ‘Resilience

as positive coping appraisals: testing the schematic appraisals model of suicide (SAMS)’,

*Behavior Research and Therapy*, vol. 48, no. 3, pp. 179-186.

54. Landerman, R, George, L, Campbell, R & Blazer, D 1989, ‘Alternative models of the

stress buffering hypothesis’, *American Journal of Community Psychology*, vol. 17, no. 5,

pp. 625-642.

55. Wardian, J, Robbins, D, Wolfersteig, W, Johnson, T & Dustman, P 2012, ‘Validation of

the DSSI-10 to measure social support in a general population’, *Research on Social Work*

*Practice*, vol. 23, no. 1, pp. 100-106.

1. Hays, R & DiMatteo, R 2010, ‘A short-form measure of loneliness’, *Journal of Personality Assessment*, vol. 51, no. 1, pp. 69-81.
2. Cohen, J, Cohen, P, West, S & Aiken L 2003, *Applied multiple regression/correlation analysis for the behavioral sciences.* Mahwah, NJ: Erlbaum.
3. Hammond, C 2004, ‘Impacts of lifelong learning upon emotional resilience, psychological and mental health: fieldwork evidence’, *Oxford Review of Education*, vol. 30, no. 4, pp. 550-568.
4. Joo, J, Hwang, S, Abu, H & Gallo J 2016, ‘An innovative model of depression care delivery: peer mentors in collaboration with a mental health professional to relieve depression in older adults’, *The American Journal of Geriatric Psychiatry*, vol. 24, no. 5, pp. 407-416.
5. Cuijpers P. Psychological outreach programmes for the depressed elderly: a meta-analysis of effects and dropout. International Journal of Geriatric Psychiatry. 1998;13(1):41-48.
