# Supplementary material for: Effectiveness of peer counseling, social engagement, and combination interventions in improving depressive symptoms of community-dwelling Filipino senior citizens
Source: PLoS One. 2020 Apr 1;15(4):e0230770. doi: 10.1371/journal.pone.0230770 (PMC7112231; doi:10.1371/journal.pone.0230770)
Supplement: S3 File — (PDF) [file pone.0230770.s003.pdf]

Graduate School of Medicine and Faculty of Medicine
The University of Tokyo
7-3-1 Hongo, Bunkyo-ku, Tokyo 113-0033, Japan

Ethics Committee

Date: October 15, 2019

Serial Number: 11641-(1)

Title of research: Project ENGAGE: An action research towards improving the psychological well-being of community-dwelling seniors in the Philippines

Name of applicant: Masamine Jimba, Professor,
Department of Community and Global Health,
Graduate School of Medicine, The University of Tokyo

This is to certify that a plan for the research project identified above was amended, reviewed, and was approved by the Ethics Committee on March 15, 2018.

THE UNIVERSITY OF TOKYO

Nobuhito Saito, Dean
Graduate School of Medicine and
Faculty of Medicine
The University of Tokyo

NS/ka

東京大学
THE UNIVERSITY OF TOKYO
