# Supplementary material for: Effectiveness of peer counseling, social engagement, and combination interventions in improving depressive symptoms of community-dwelling Filipino senior citizens
Source: PLoS One. 2020 Apr 1;15(4):e0230770. doi: 10.1371/journal.pone.0230770 (PMC7112231; doi:10.1371/journal.pone.0230770)
Supplement: S4 File — (PDF) [file pone.0230770.s004.pdf]

University of the Philippines Manila
RESEARCH ETHICS BOARD
2nd Floor Paz Mendoza Building, College of Medicine, UP Manila
547 Pedro Gil Street, Ermita, 1000 Manila
Telephone: +63 2 5264346; *Mobile*: +63 927 3264910; *Email*: upmreb@post.upm.edu.ph

19 March 2018

MR. ROGIE ROYCE CARANDANG
Principal Investigator
University of Tokyo

Re: UPMREB 2017-312-01
Project ENGAGE: An action research towards improving the psychological wellbeing of community-dwelling seniors in the Philippines

Dear **MR. CARANDANG:**

We wish to inform you that the **UP Manila Research Ethics Board (UPMREB) Review Panel 1** approved the proposed amendment/s in your study entitled, "**Project ENGAGE: An action research towards improving the psychological wellbeing of community-dwelling seniors in the Philippines" (UPMREB 2017-312-01)** during its meeting on 13 March 2018. Upon review of UPMREB FORM3(A)2012: Study Protocol Amendment Submission Form and attachments, the following documents have been approved for use:

- Study protocol version 12 dated 07 February 2018

Thank you.

Very truly yours,

JACINTO BLAS V. MANTARING III, MD, MSc
Chair, UPMREB Review Panel 1
